# C3aR-initiated signaling is a critical mechanism of podocyte injury in membranous nephropathy

**DOI:** 10.1172/jci.insight.172976

**Published:** 2024-01-16

**Authors:** Qi Zhang, Sofia Bin, Kelly Budge, Astgik Petrosyan, Valentina Villani, Paola Aguiari, Coralien Vink, Jack Wetzels, Hasmik Soloyan, Gaetano La Manna, Manuel Alfredo Podestà, Paolo Molinari, Sargis Sedrakyan, Kevin V. Lemley, Roger E. De Filippo, Laura Perin, Paolo Cravedi, Stefano Da Sacco

**Affiliations:** 1 GOFARR Laboratory for Organ Regenerative Research and Cell Therapeutics in Urology, Saban Research Institute, Division of Urology, Children’s Hospital Los Angeles (CHLA), Los Angeles, California, USA.; 2 Translational Transplant Research Center and Renal Division, Department of Medicine, Icahn School of Medicine at Mount Sinai, New York, New York, USA.; 3 Nephrology, Dialysis and Renal Transplant Unit, IRCCS - Azienda Ospedaliero-Universitaria di Bologna, Alma Mater Studiorum University of Bologna, Bologna, Italy.; 4 Department of Urology, Keck School of Medicine, University of Southern California, Los Angeles, California, USA.; 5 Department of Nephrology, Radboud University Medical Center, Nijmegen, The Netherlands.; 6 Transplantation Research Center, Renal Division, Brigham and Women’s Hospital, Harvard Medical School, Boston, Massachusetts, USA.

**Keywords:** Nephrology, Chronic kidney disease, Complement

## Abstract

The deposition of antipodocyte autoantibodies in the glomerular subepithelial space induces primary membranous nephropathy (MN), the leading cause of nephrotic syndrome worldwide. Taking advantage of the glomerulus-on-a-chip system, we modeled human primary MN induced by anti-PLA2R antibodies. Here we show that exposure of primary human podocytes expressing PLA2R to MN serum results in IgG deposition and complement activation on their surface, leading to loss of the chip permselectivity to albumin. C3a receptor (C3aR) antagonists as well as C3AR gene silencing in podocytes reduced oxidative stress induced by MN serum and prevented albumin leakage. In contrast, inhibition of the formation of the membrane-attack-complex (MAC), previously thought to play a major role in MN pathogenesis, did not affect permselectivity to albumin. In addition, treatment with a C3aR antagonist effectively prevented proteinuria in a mouse model of MN, substantiating the chip findings. In conclusion, using a combination of pathophysiologically relevant in vitro and in vivo models, we established that C3a/C3aR signaling plays a critical role in complement-mediated MN pathogenesis, indicating an alternative therapeutic target for MN.

## Abstract 2

C3a/C3aR signaling mediates podocyte injury in primary membranous nephropathy, and inhibtion of this pathway prevents proteinuriai n a mouse model.

## Introduction

Membranous nephropathy (MN), one of the leading causes of nephrotic syndrome in adults, is an immune-mediated glomerular disorder characterized by the subepithelial deposition of autoantibodies targeting podocyte antigens. While phospholipase A2 receptor (PLA2R) has been identified as the major target antigen in approximately 70% of patients with MN (1–3), autoantibodies targeting other antigens, including thrombospondin (THSD7A), Sema3B, and PCDH7 (4), can be found in the remaining cases (4, 5).

Binding of these autoantibodies to their podocyte target antigens initiates pathogenic mechanisms that initiate podocyte injury and loss of glomerular permselectivity, leading to loss of kidney function (6). These events have been traditionally regarded as effects mediated by the activation of the complement cascade (4, 6–12) and its activation of the membrane attack complex (MAC). However, data on the role of MAC as the sole driver of podocyte injury are still conflicting (11, 12) and recent studies point at complement receptor-initiated mechanisms (11, 12) as a possible player in MN. Therefore, understanding the complement effector mechanisms is a relevant issue, as different complement-targeting agents could possibly be used in clinical settings to specifically target these effectors.

Here, we studied the pathogenic effects of anti-PLA2R antibodies on the glomerular filtration barrier (GFB) using our validated human glomerulus-on-a-chip (GOAC) platform (13) and further validated our results in an in vivo mouse model of MN mice. This MN mouse model was generated by injection of serum from patients with MN with autoantibodies against THSD7A (14), an antigen that is expressed by murine podocytes since PLA2R is not expressed in mice (14). Our studies showed that PLA2R expression in human podocytes is essential for injury initiation in the GOAC and that both IgG and complement are required to initiate podocyte damage and lead to albumin leakage in vitro. Furthermore, our in vitro studies suggest that MAC formation may not be the sole effector mechanism of complement-mediated podocyte injury in MN. We also proved that activation of the C3a/C3aR pathway plays a critical role in the disruption of the GFB both in vitro and in vivo, leading to changes in podocyte phenotype and function. Finally, we show that administration of a C3aR antagonist and *C3ar* gene silencing can preserve permselectivity both in our in vitro system and in the animal model.

In summary, we have established that C3a/C3aR signaling plays a critical role in complement-mediated MN pathogenesis, indicating a potentially novel therapeutic target for MN.

## Results

### PLA2R expression on podocytes is necessary for injury induction by anti-PLA2R^+^ MN sera.

**Figure 1:**
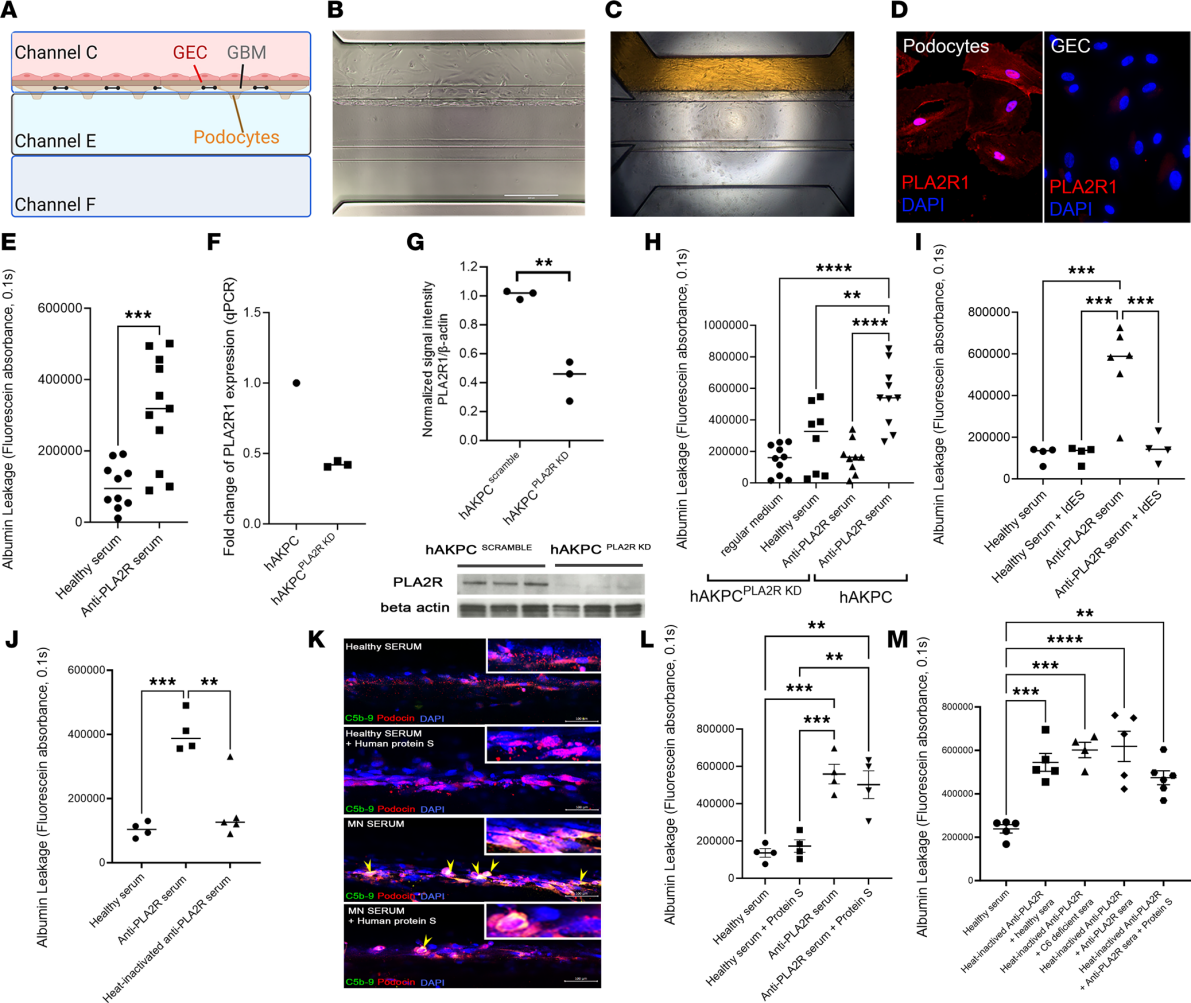
The GOAC recapitulates podocyte injury in vitro. (**A**) Schematic representation of the GOAC. (**B**) Light microscope image, depicting the formation of the barrier (72 hours). Scale bar: 400 µm. (**C**) Representative image of albumin test (albumin-FITC, 40 mg/mL, yellow) confirming permselectivity. (**D**) Podocytes in vitro express PLA2R (red). Scale bar: 25 µm. DAPI, blue. (**E**) Measurement of FITC-albumin in GOAC exposed to anti-PLA2R MN serum (*n* ≥ 4/group). Additional results in Supplemental Figure 2. (**F**) PLA2R1 RNA expression following knockdown on hAKPC cells; control: scramble hAKPC cells. (**G**) Western Blotting showing reduced PLA2R (150 kDa) expression after KD in hAKPC-derived podocytes. β-Actin: 42 KDa; *n* = 3/group. (**H**) Measurement of FITC-albumin in GOAC filtrate with PLA2R–knocked-down hAKPC-derived podocytes (groups: regular media, healthy serum and PLA2R-Ab+ MN serum, *n* ≥ 4/group.) (**I**) Antibody neutralization by IdeS in MN serum prevents GOAC leakage (*n* ≥ 4/group). Additional results in Supplemental Figure 5. (**J**)Complement neutralization by heat-inactivation successfully prevents GOAC injury by MN patient serum (*n* ≥ 4/group). Additional results in Supplemental Figure 6. (**K**) C5b-9 (MAC) formation on GOAC after serum exposure. Healthy serum with or without protein S (no MAC, top 2 panels) vs. MN serum (MAC, third panel) in podocin^+^ cells (red). Protein S prevents MAC formation (lower panel). Yellow arrows: MAC/podocin overlap. Magnified views of GEC (unstained) and podocin^+^ cells (red) with or without MAC (green); DAPI, blue. Scale bar: 100 µm. (**L**) MAC neutralization, while preventing C5b-9 formation, does not prevent leakage (*n* = 4/group). Additional results in Supplemental Figure 6. (**M**) GOAC exposure to healthy serum (1%), heat-inactivated anti-PLA2R (0.5%) + healthy serum (0.5%), heat-inactivated anti-PLA2R (0.5%) + C6-deficient healthy serum (0.5%), heat-inactivated- anti-PLA2R (0.5%) + anti-PLA2R (0.5%), or heat-inactivated anti-PLA2R (0.5%) + anti-PLA2R (0.5%) + protein S. (*n* ≥ 4/group). All statistical values determined by 1-way ANOVA with the exclusion of **E** and **G** (2-tailed Student’s *t* test). *P < 0.05 **P < 0.01; ***P < 0.001; ****P < 0.0001.

Figure 1, A–C, summarizes the structure of GOAC (13, 15), generated using human primary podocytes (hPOD) and human glomerular endothelial cells (hGEC; Figure 1, A–C, and Supplemental Figure 1, A–F; supplemental material available online with this article; https://doi.org/10.1172/jci.insight.172976DS1). First, we report the specificity of expression of PLA2R on hPOD (Figure 1D and Supplemental Figure 1) but not in hGEC. In this system, serum from patients with anti-PLA2R^+^ MN promotes a statistically significant loss of permselectivity documented by increased albumin leakage (Figure 1E and Supplemental Figure 2). We also confirmed that exposure to anti-PLA2R serum is accompanied by an increased presence of C3b and C5b-9 as confirmed by immunofluorescence (Supplemental Figure 3), thus validating the use of our system to study the complement cascade in the GOAC. Next, to test the pathogenic role of anti-PLA2R antibodies, we silenced the PLA2R gene in podocytes. Since transfection protocols require extensive in vitro culture, silencing of *PLA2R* was performed using human amniotic fluid kidney progenitor cells (16) (hAKPC^PLA2R^ ^KD^) instead of hPOD. To note, we have previously confirmed that response of hAKPC-P to MN serum is similar to hPOD (13). After validation of efficient silencing (Figure 1, F and G), GOAC knockdown (KD-GOAC) were set up as previously described (13, 15). We detected that albumin leakage was prevented in GOAC generated with hAKPC^PLA2R^ ^KD^ compared with normal podocytes (Figure 1H). Notably, silencing of PLA2R1 in hGEC alone did not prevent albumin leakage in the GOAC (Supplemental Figure 4), suggesting that binding of anti-PLA2R autoantibodies to their target antigen PLA2R specifically on podocytes exerts a pathogenic role on the in vitro GOAC, affecting its permselectivity.

### Albumin leakage induced by MN sera in the GOAC requires both IgG and an intact complement system.

Glomerular deposition of IgG4 and activation of the complement alternative pathway (17) has been shown to occur in primary MN. To test the importance of IgG integrity in mediating glomerular damage, we treated healthy and MN sera with IgG-cleaving endopeptidase (IdeS), before exposure to the chips. This enzyme cleaves the Fc portion of IgG, preventing their complement-activating function (18) (Supplemental Figure 5, A–C). While healthy serum treated with IdeS did not exhibit any change in albumin leakage (indicating the absence of deleterious effects caused by IdeS), preexposure of human MN sera to IdeS prevented albumin leakage (Figure 1I and Supplemental Figure 5, D and E), confirming that the increased permeability induced by MN serum requires preserved IgG structure.

Activation of the complement cascade and formation of the MAC have been considered key steps in the induction of podocyte injury in MN (19). Indeed, human sera that underwent heat inactivation of complement were unable to trigger detectable injury in the GOAC (Figure 1J and Supplemental Figure 6, B and C), as confirmed by the preservation of permselectivity, supporting the role of complement in promoting damage to the GOAC. To specifically assess the relevance of MAC formation in GFB disruption, we added recombinant protein S (soluble vitronectin), which inhibits MAC formation by preventing C5b-7 assembly and C9 polymerization (20), to GOAC exposed to anti-PLA2R^+^ MN sera. While the addition of protein S efficiently inhibited the formation of MAC on podocytes, as confirmed by Western blotting (Supplemental Figure 7A) and immunofluorescence (Figure 1K), albumin leakage was only marginally decreased (no statistical significance; Figure 1L and Supplemental Figure 7, B and C). Results were also confirmed using a combination of heat-inactivated anti-PLA2R sera and C6-deficient sera as shown in Figure 1M, suggesting that alternative or additional mechanisms of damage are triggered by anti-PLA2R binding to podocytes.

### C3a/C3aR signaling mediates MN-induced injury in the GOAC.

We have previously shown that ligation of C3a to its receptor C3aR on cultured podocytes induces actin cytoskeleton rearrangement and cell damage (21).

**Figure 2:**
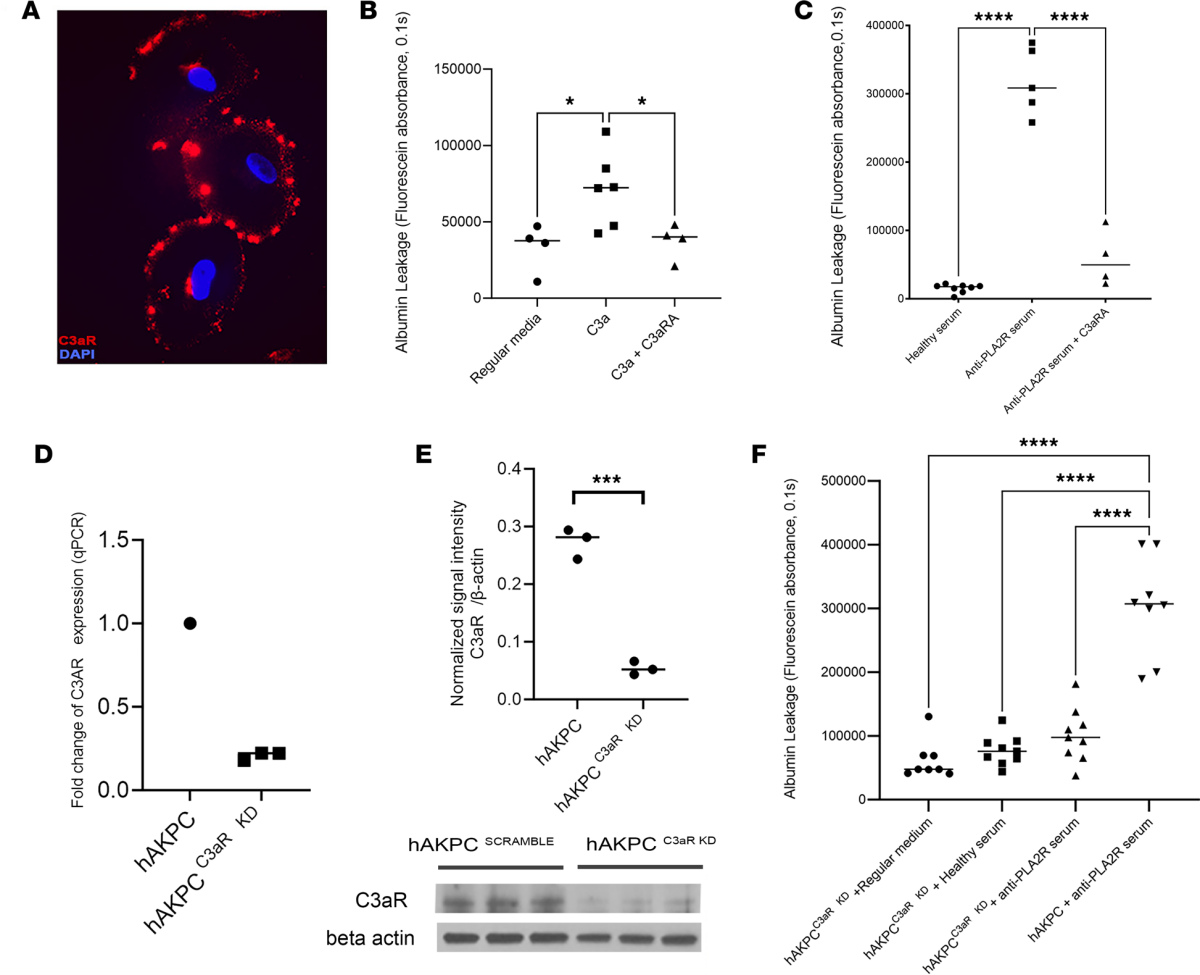
C3aR/C3aR signaling plays a key role in PLA2R^+^ MN-induced injury. (**A**) Representative picture of immunofluorescence staining confirming expression of C3aR (red, top panel) in podocytes in vitro. DAPI nuclear staining: blue. Magnification, ×20. (**B**) Box plot graph of fluorescein absorbance (485 nm/535 nm, 0.1 second) in filtrate collected from channel F 60 minutes after addition of albumin. Exposure of GOAC to 50 nM C3a for 24 hours increased albumin leakage compared with the control group. Exposure to the chip to 50 nM C3aR antagonist was able to prevent the deleterious C3a effect, confirming the role of C3a in driving injury in the GOAC. *P < 0.05, *n* ≥ 4/group. (**C**) Box plot graph of fluorescein absorbance (485 nm/535 nm, 0.1 second) in filtrate collected from channel F 60 minutes after addition of albumin. Addition of 50 nM C3aR antagonist to GOAC was able to prevent leakage caused by MN serum, further confirming the involvement of C3a in podocyte damage. ****P < 0.0001, *n* ≥ 4/group. (**D**) Measurement of C3ar RNA expression by qPCR in control hAKPC-derived podocytes and hAKPC-derived podocytes in which C3ar was knocked down, confirming an 80% decrease in its expression. *n* = 3, Ctrl hAKPC; *n* = 3, KD hAKPC. (**E**) Western blotting analysis of C3aR in hAKPC-derived podocytes and hAKPC-derived podocytes in which C3ar was knocked down confirmed an efficient decrease in protein expression. Measured density for C3aR bands (54 KDa) was normalized against β-actin, showing a significantly decreased protein level. ***P < 0.001. β-Actin: 42 KDa; *n* = 3/group. (**F**) Box plot graph of fluorescein absorbance (485 nm/535 nm, 0.1 second) in filtrate collected from channel F 60 minutes after addition of albumin confirming a statistically significant decrease in MN sera–induced albumin leakage in GOAC generated with hAKPC^C3aR^ ^KD^ podocytes compared with control. ****P < 0.0001; *n* ≥ 4/group. All statistical values determined by 1-way ANOVA, with the exclusion of **E** , which used 2-tailed Student’s *t* test.

Following confirmation that hPOD express C3aR (21) (Figure 2A and Supplemental Figure 7D), we stimulated chips with recombinant C3a (50 nM) for 24 hours, which induced albumin leakage, an effect that was rescued by the addition of C3aR antagonist to the media (Figure 2B). Similarly, C3aR antagonist prevented albumin leakage following exposure to MN sera, further supporting the pathogenic role of C3a/C3aR signaling in GFB dysfunction on the chip (Figure 2C). Moreover, ELISA assay confirmed that C3a is generated by the cells within the system following exposure to MN serum (Supplemental Figure 8A).

To further confirm the critical role of C3a/C3aR signaling in MN injury, we silenced *C3aR* in hAKPC (60%–80% reduction; Figure 2, D and E) and generated GOAC with either hAKPC or hAKPC^C3aR KD^. Notably, silencing of C3aR in podocytes resulted in prevention of albumin leakage upon exposure to MN serum (Figure 2F). Exposure of GOAC to C5a did not lead to permselectivity loss (Supplemental Figure 8B); similarly, addition of C5aR antagonist to GOAC exposed to MN serum did not prevent albumin leakage (Supplemental Figure 8C), further supporting a specific role for C3a/C3aR signaling as a mediator of podocyte injury.

### MN serum changes the phenotype of podocytes in vitro.

**Figure 3:**
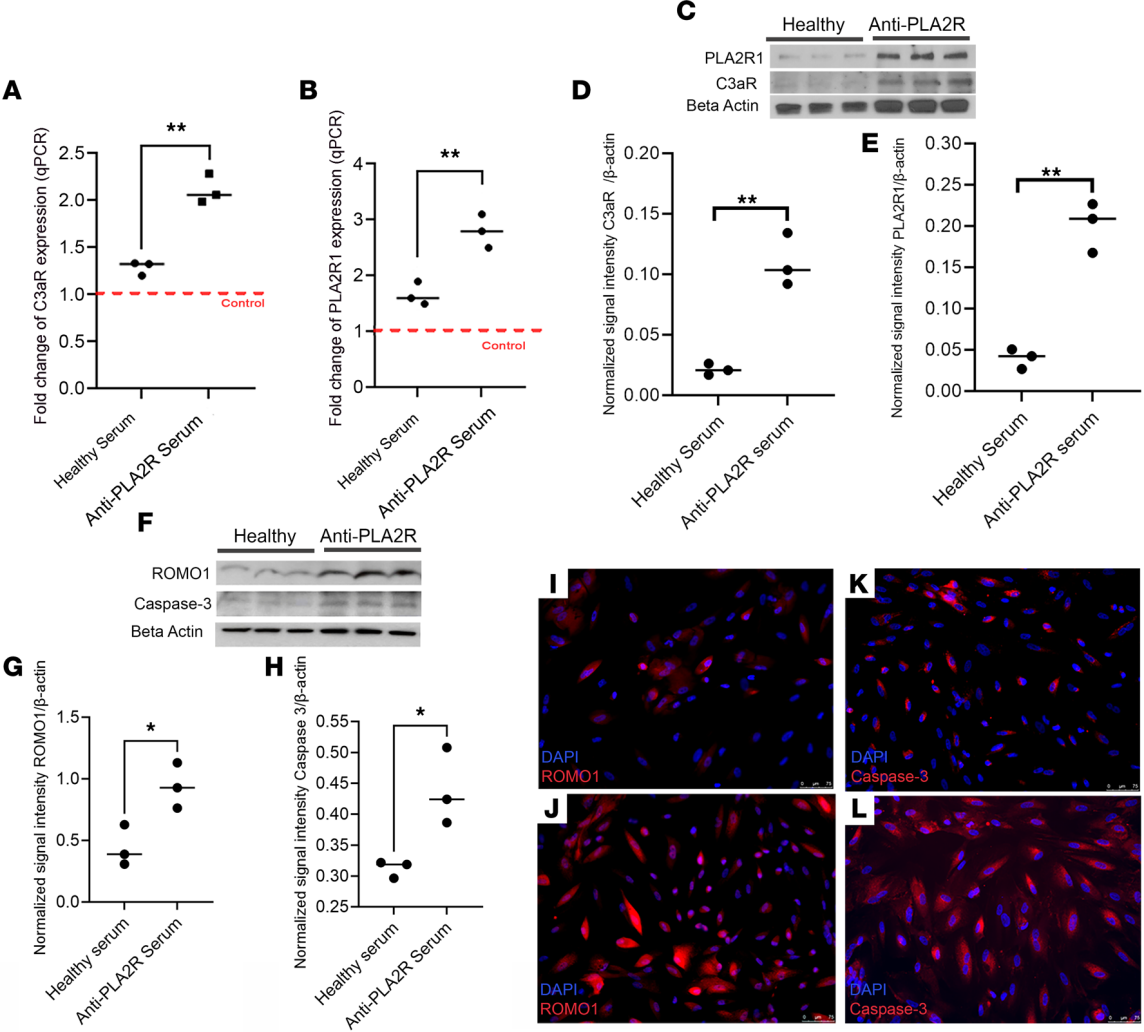
Effect of anti-PLA2R serum on podocyte phenotype. (**A** and **B**) C3aR (**A**) and PLA2R1 (**B**) RNA expression in podocytes exposed to 0.5% healthy or MN serum for 72 hours. Red dotted line: hPOD in media with no serum, normalization group (*n* = 3/group). (**C**) Western Blotting bands for C3aR (54kDa) and PLA2R1 (150 kDa) and respective beta actin (42 kDa) exposed to healthy or anti-PLA2R serum (*n* = 3/group). (**D**) Western blotting analysis of C3aR (54kDa) in hPOD monolayers exposed to MN sera, confirming an increased expression after 72 hours. Bands were normalized against β-actin (42 kDa), showing a significantly increased expression. *n* = 3/group. (**E**) Western blotting analysis of PLA2R (150 kDa) in hPOD monolayers exposed to MN sera, showing a statistically significant increase in protein expression at 72 hours. Bands were normalized against β-actin (42 kDa), showing a significantly increased expression (*n* = 3/group). (**F**) Western Blotting bands for ROMO1 (10 kDa) and caspase-3 (35 kDa) and respective β-actin (42 kDa) exposed to healthy or anti-PLA2R serum (*n* = 3/group). (**G**) Western blotting analysis of oxidative stress, measured by expression of ROMO1 (10 kDa), in hPOD monolayers exposed to MN sera. Bands were normalized against β-actin (42 kDa), showing a significantly increased protein level (*n* = 3/group). (**H**) Western blotting analysis of apoptosis, measured by expression of caspase-3 (35 kDa), in hPOD monolayers exposed to MN sera. Bands were normalized against β-actin (42 kDa), showing an increased expression (*n* = 3/group). (**I** and **J**) hPOD monolayers exposed to healthy (**I**) or MN serum (**J**) confirmed an increase in ROMO1 (red). DAPI, blue. Scale bar: 25 µm. (**K** and **L**) Representative immunofluorescence staining of hPOD monolayers exposed to healthy (**K**) or MN serum (**L**) confirmed an increase in caspase-3 (red). DAPI, blue. Scale bar: 25 µm. All statistical values determined by 2-tailed Student’s *t* test. *P < 0.05, **P < 0.01.

To understand the pathogenic mechanisms of the MN serum on podocytes, we exposed primary cells to 0.5% MN serum containing anti-PLA2R antibodies for 72 hours. Expression of C3aR and PLA2R mRNA and protein significantly increased (Figure 3, A–E), an effect that was not observed after exposure to sera from healthy controls. Exposure of GOAC to MN sera stimulated both podocyte oxidative stress and apoptosis, as shown by heightened levels of ROMO1 (Figure 3, F, G, I, and J), known to increase in response to oxidative stress events (22) and caspase-3 (Figure 3, F, H, K, and L), which is involved in apoptotic pathways (23). Taken together, these data further confirm previous reports that MN serum affects podocytes by modifying their phenotype and function.

### C3aR antagonist exerts a protective effect on podocytes in vitro.

**Figure 4:**
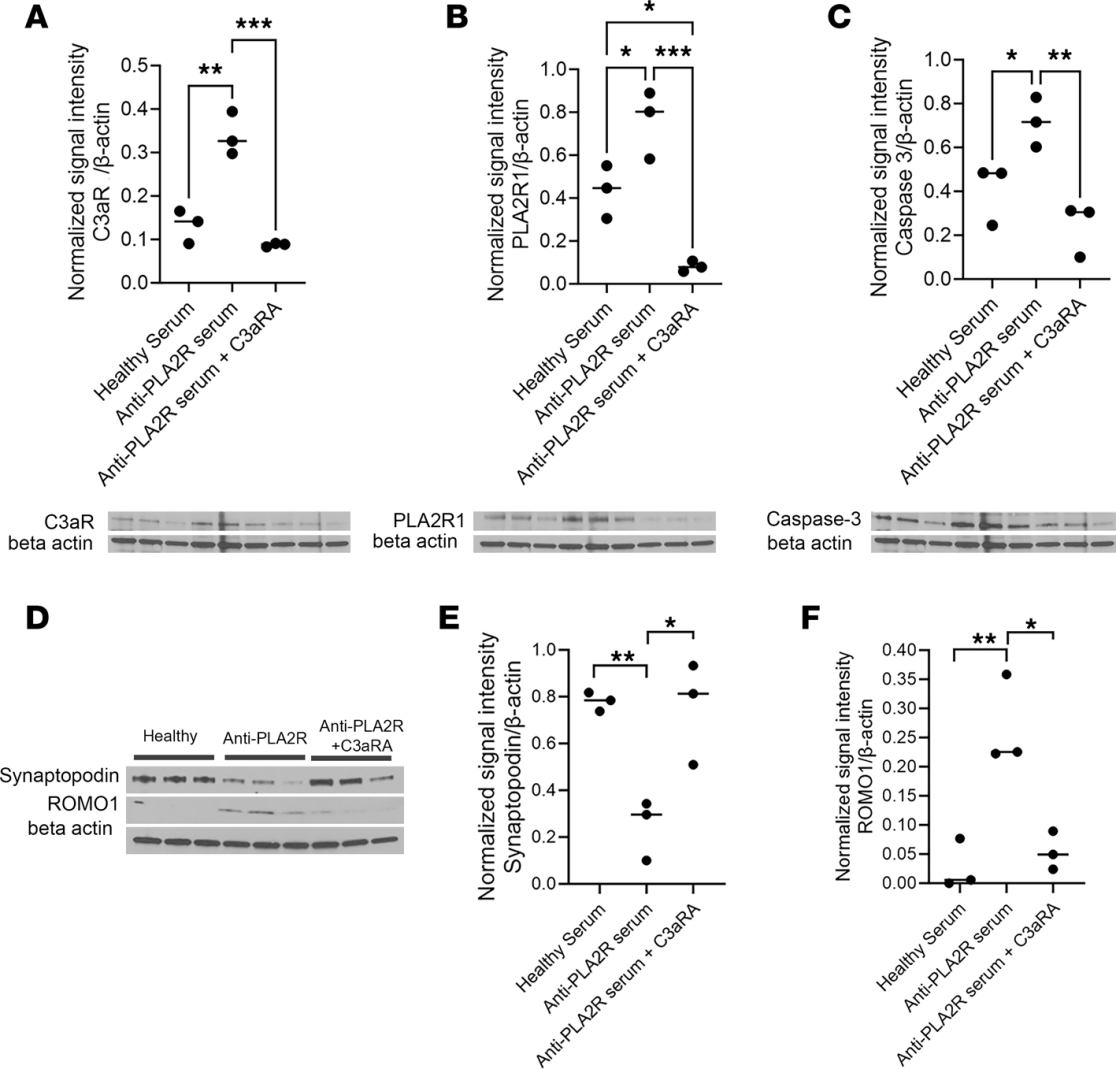
C3aR antagonism effectively prevents changes in podocyte phenotype by MN serum. (**A**) Western blotting analysis of C3aR in hPOD monolayers exposed to MN sera or MN sera + C3aRA. The antagonist efficiently prevented C3aR (54 kDa) increase. Bands were normalized against β-actin (42 KDa), showing a significantly decreased protein level. *n* = 3/group. (**B**) Western blotting analysis of PLA2R (150kDa) in hPOD monolayers exposed to MN sera or MN sera + C3aRA. The antagonist efficiently prevented PLA2R increase. Bands were normalized against β-actin (42 kDa), showing a significantly decreased protein level. *n* = 3/group. (**C**) Western blotting analysis of apoptosis, measured by expression of caspase-3 (35 kDa), in hPOD monolayers exposed to MN sera or MN sera + C3aRA. The antagonist significantly decreased apoptosis in hPOD. Bands were normalized against β-actin (42 kDa), showing a significantly decreased protein level. *n* = 3/group. (**D**) Western Blotting bands for Synaptopodin (100kDa) and ROMO1 (10 kDa) and respective β-actin (42 kDa) after exposure to healthy serum, anti-PLA2R serum, or anti-PLA2R serum + C3aRA. (*n* = 3/group). (**E**) Western blotting analysis of synaptopodin (100 kDa) in hPOD monolayers exposed to MN sera or MN sera+ C3aRA. The antagonist efficiently prevented synaptopodin loss. Bands were normalized against β-actin (42 kDa), showing a significantly increased protein level. Bands are shown in **D**. *n* of replicates/group: 3. (**F**) Western blotting analysis of oxidative stress, measured by expression of ROMO1 (10 kDa), in hPOD monolayers exposed to MN sera or MN sera+ C3aRA. The antagonist significantly decreased ROMO1 expression in hPOD. Bands were normalized against β-actin (42 kDa), showing a significantly decreased protein level. *n* = 3/group. Bands are shown in **D**. All statistical values determined by 1-way ANOVA. *P < 0.05; **P < 0.01, ***P < 0.001.

To test whether changes detected on podocytes were modulated by the C3a/C3aR signaling axes, we repeated the experiments by exposing the cells to MN serum with or without a C3aR antagonist (Figure 4). The C3aR antagonist significantly prevented increases in C3aR and PLA2R protein expression, confirming previous results by Gao et al. (12) (Figure 4, A and B). It also successfully prevented the loss of synaptopodin, an actin-associated protein essential for the podocyte cytoskeleton function (24) (Figure 4, A and B). Inhibition of the C3a/C3aR signaling cascade was also able to avert oxidative stress and apoptosis in podocytes, as shown by protein expression analysis for ROMO1 and caspase-3 (Figure 4, D and E, and Supplemental Figure 9A). When the same experiment was performed on podocytes not exposed to MN sera, we confirmed a decrease in the expression of PLA2R1 as well as caspase-3 but not C3aR, suggesting that the activation of C3aR is indeed modulating PLA2R in podocytes (Supplemental Figure 9).

### C3aR antagonism prevents the MN severity in vivo.

**Figure 5:**
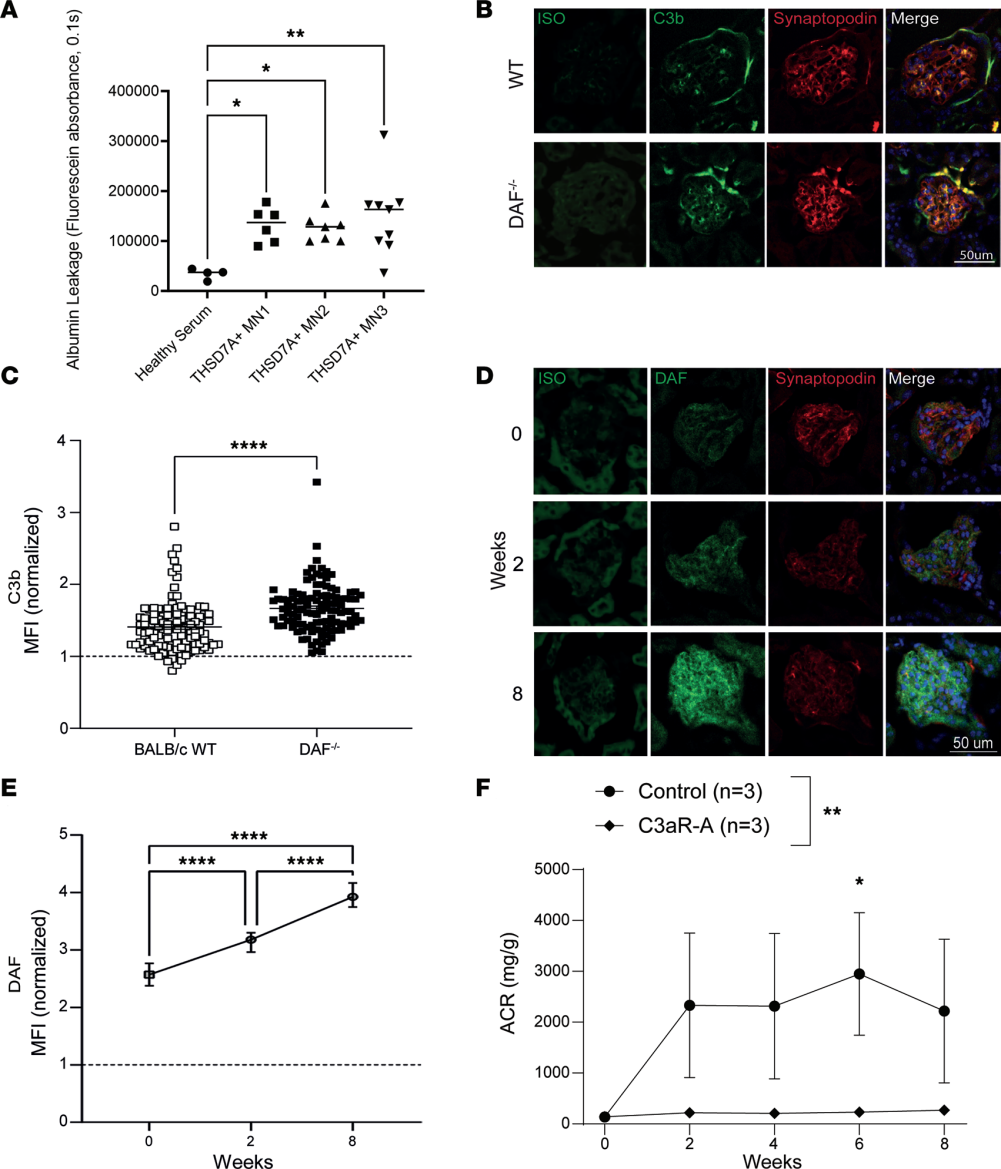
DAF expression and C3a/C3aR signaling affect disease severity in murine anti-THSD7a antibody–induced MN. (**A**) Box plot graph of fluorescein absorbance (485 nm/535 nm, 0.1 second) in filtrate collected from channel F 60 minutes after addition of albumin, confirming a statistically significant increase in anti-THSD7A^+^ sera–induced albumin leakage in GOAC compared with control. Statistical values determined by 1-way ANOVA. *P < 0.05; **P < 0.01; *n* ≥ 4/group. (**B**) C3b and synaptopodin staining in representative glomeruli from WT and *DAF*^–/–^ mice at 8 weeks after injection of serum from patients with MN with anti-THSD7a antibodies. Scale bar: 50 μm. (**C**) Quantification of C3b deposition (BALB/c WT, *n* = 3; *DAF*^–/–^, *n* = 3). Glomerular fluorescence intensity was quantified measuring MFI in all glomeruli of a kidney section. DAF and C3b expression was then divided by the average MFI of glomeruli stained with isotype (normalized data) using ImageJ software. Statistical values determined by Mann-Whitney *U* test. ****P < 0.0001. (**D**) DAF and synaptopodin staining in representative glomeruli from WT mice at 0, 2, and 8 weeks after injection of serum from patients with MN with anti-THSD7a antibodies. Scale bar: 50 μm. (**E**) Quantification of DAF immunofluorescence (0 weeks, *n* = 3; 2 weeks, *n* = 2; 8 weeks, *n* = 2). Glomerular fluorescence intensity was quantified relatively to isotype using ImageJ software. Statistical values determined by Kruskal-Wallis test. ****P < 0.0001. (**F**) Urinary albumin/creatinine (ACR) levels in *DAF*^–/–^ BALB/c male mice injected with serum from patients with MN with anti-THSD7a antibodies. One group of mice was treated with C3aR antagonist starting from the day of serum injection (THSD7a sera, *n* = 3; THSD7a sera + C3aRA, *n* = 3). Statistical values determined by 2-way ANOVA. *P < 0.05, **P < 0.001.

It is known that mice do not express PLA2R,making these mice challenging for modeling MN. However, THSD7A, another target antigen in a minority of human MN cases, is expressed by murine podocytes and can be targeted to mimic MN (14). Notably, exposure of GOAC containing THSD7A-expressing podocytes (Supplemental Figure 10) to human anti-THSD7A serum elicited albumin leak (Figure 5A) similarly to the anti-PLA2R sera (Figure 2). Based on this observation, we tested whether the same mechanisms identified in vitro apply also in vivo in mice. We injected male BALB/c mice with heat-inactivated serum from 5 patients with MN with anti-THSD7A antibodies (see Supplemental Table 1 for their characteristics). In this model, human IgG bind to murine THSD7a in the glomeruli, and murine IgG specific for human IgG form immune complexes in the subepithelial space of the glomeruli (Supplemental Figure 11) that activate complement and mediate podocyte injury.

Despite murine IgG deposition in the glomeruli already at 1 week after injection (Supplemental Figure 11E), we did not observe significant albuminuria (Supplemental Figure 12). Since complement represents the main effector mechanism in MN, we stained kidneys from anti-THSD7a antibody serum–injected mice for C3b, which was extremely faint (Figure 5, B and C).

Therefore, we reasoned that the lack of complement activation in vivo despite murine IgG deposition in the glomeruli could be induced by upregulation of complement inhibitors. Decay-accelerating factor (DAF) is a membrane-bound complement regulator expressed on podocytes’ membranes that accelerates decay of C3 convertase, preventing downstream complement cascade activation, such as the formation of C3, C5a, and MAC assembly (21). When we stained glomeruli for DAF expression, we found a significant upregulation at 2 and 8 weeks after anti-THSD7a serum injection (Figure 5, D and E), thus preventing complement activation, as indicated by limited C3b deposition. We found that *DAF* gene is similarly upregulated in the glomeruli of patients with MN (Supplementary Supplemental Figure 13).

Therefore, to enhance complement activation in this model and increase podocyte injury, we injected anti-THSD7a^+^ sera into mice with *Daf* gene deletion, as a strategy to locally activate complement. *DAF*^–/–^ mice do not display any kidney abnormality at baseline (21). However, upon injection with serum from patients with anti-THSD7a antibodies, *DAF*^–/–^ mice displayed clear C3b deposition (Figure 5, B and C).

**Figure 6:**
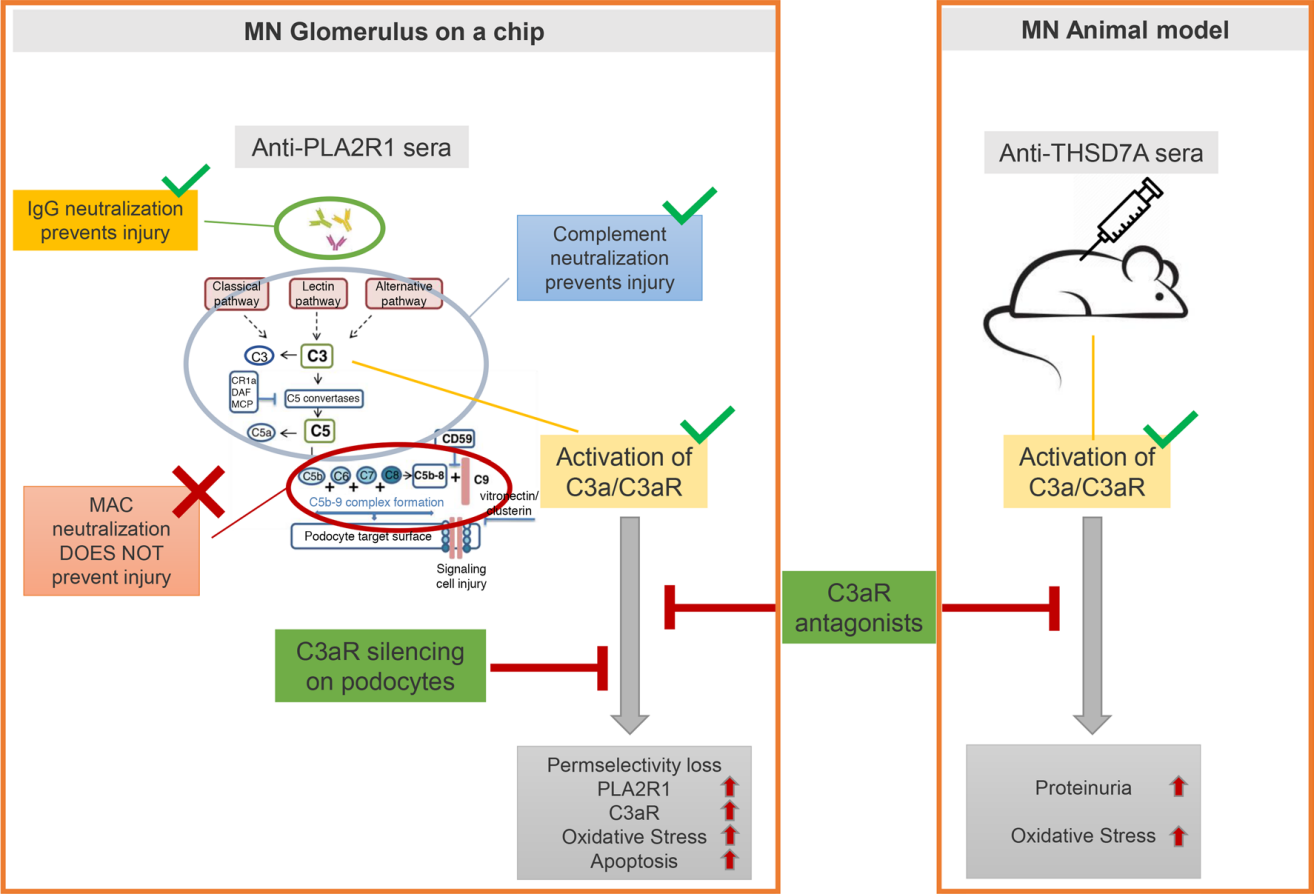
Working model. Representative scheme of the working model for the presented work.

We then tested the hypothesis that a C3aR antagonist prevents anti-THSD7a antibody–induced MN in vivo. We administered C3aR antagonist to *DAF*^–/–^ mice 24 hours before the injection with serum from patients with MN with anti-THSD7A antibodies. Our data (Figure 5F) show that, while untreated *DAF*^–/–^ mice developed severe albuminuria, disease was fully prevented, as shown by the lack of proteinuria in the cohort treated with C3aRA. Altogether, these data indicate that DAF prevents complement activation in anti-THSD7a antibody–induced MN in mice and that C3a/C3aR signaling is a key mediator of podocyte injury in this model (Figure 6).

## Discussion

In MN, complement activation has been recognized as the driving force leading to glomerular damage and proteinuria, but effector mechanisms have not been clearly defined (4). MAC formation has been considered a key pathogenic mechanism (4, 6), but outcomes in experimental studies have shown contrasting results (7–9). More recently, it has been suggested that both MAC assembly and C3aR and C5aR1 signaling are required to induce proteolysis of synaptopodin and NEPH1 proteins in podocytes (11). Gao et al. showed that C3a from the plasma of patients with MN reduced the expression of synaptopodin, impaired podocyte migration function, and decreased cell viability and that these effects were rescued by C3aR antagonism (12). These studies were performed using immortalized podocyte cell lines in which the expression of PRL2A was not clearly confirmed and may not fully recapitulate the features of primary cells (13, 16). Additionally, the use of podocyte monocultures does not truly recapitulate the GFB, and the in vitro studies published so far do not show functional readout (13, 15, 16).

Furthermore, although hypothesized since the first description of anti-PLA2R antibodies, formal proof of their pathogenic role in MN has been only recently obtained (25). In the current study, we have mechanistically demonstrated that binding of intact anti-PLA2R autoantibodies to podocytes and complement activation are necessary to elicit the cascade of events that ultimately lead to functional impairment in a MN setting modeled in vitro.

We also confirmed that activation of the complement system is necessary to induce injury and loss of permselectivity in this context. Interestingly, at variance with data obtained from immortalized podocyte cultures (11), inhibition of MAC assembly did not prevent albumin leakage in the GOAC system, suggesting that short-term podocyte damage in this setting is not mediated by the terminal complement complex.

Importantly, we show that suppression of the C3a/C3aR signaling pathway is sufficient to prevent podocyte damage induced by anti-PLA2R antibody ligation to their molecular target. This action directly translates into abrupt changes in the GFB of the GOAC with loss of permselectivity, confirming a functional role for the C3a/C3aR axis in our model of MN.

Our working model (Figure 6) generated using data from the GOAC system has been supported also by data obtained in vivo in mice injected with serum from patients with MN with anti-THSD7a antibodies. Binding of human IgG to THSD7a on murine podocytes is followed by an autologous phase in which the development of murine IgG targeting human anti-THSD7a antibodies locally activates the complement cascade. In this setting, C3aR antagonist was enough to prevent disease. Of note, we were able to elicit significant complement activation only using *Daf*-deficient mice, suggesting that the complement regulator DAF plays a critical role in preventing complement activation and podocyte injury (26). Binding of human IgG to podocytes alone was not enough to promote detectable albuminuria.

Upon anti-THSD7a antibody injection, podocytes upregulate DAF expression, preventing the onset of the disease. Conversely, genetic DAF deletion unleashed complement activation, leading to C3a/C3aR-mediated podocyte injury in vivo in mice. We found that DAF is similarly upregulated in humans with the disease (Supplemental Figure 13) (27). However, constant autoantibody deposition may overcome the regulatory effects of DAF, leading to complement activation and C3a formation. Intriguingly, individuals with loss-of-function mutations in *Daf* gene have proteinuria, further supporting the concept that this complement regulator is important in protecting podocytes from uncontrolled complement activation (28). The above findings confirm and extend previous data by Gao et al. (12) using a podocyte cell line genetically modified to overexpress PLA2R. In this study, increased plasma levels of C3a were also observed in a small cohort of patients with primary MN, whose kidney biopsies had higher C3b deposition on podocytes compared with healthy controls. The authors described increased levels of PLA2R and C3aR after exposure to MN sera, with decreased synaptopodin expression and podocyte migratory capability. Our data using GOAC confirm this finding and document that C3aR antagonism prevents MN serum–induced PLA2R increase. By silencing PLA2R and C3aR on podocytes, we were also able to confirm that injury is mainly driven by podocyte, not endothelial cell, damage. Intriguingly, a recent single-cell RNA-Seq study on biopsies from patients with MN showed that apoptosis was present mostly in mesangial cells (29), but our data suggest that apoptosis may play a role also in MN-mediated podocyte injury. Further studies, beyond the scope of this work, are needed to confirm its effect.

There are caveats that should be considered in interpreting our results. We recognize that the GOAC, although clinically relevant, still represents a system that does not capture all the complexities of in vivo human glomeruli. However, the fact that our in vitro findings were confirmed using an in vivo model supports the role of C3a/C3aR signaling in MN pathophysiology. In addition, we acknowledge that our studies do not fully rule out a role for C5a/C5aR1 signaling — and, possibly, MAC — but they support the concept that C3aR activation plays a dominant effector role. Even if we did not experimentally test the complement activation pathway leading to complement injury, we speculate that it is likely that binding of aberrantly glycosylated anti-PLA2R IgG4 to PLA2R on podocytes leads to complement activation through the lectin activation pathway, as shown by Haddad et al. (11).

Altogether, our data provide evidence that anti-PLA2R antibodies exert complement-mediated injury to podocytes. In light of the growing number of complement-targeting therapeutic molecules (30), these data set the basis for clinical studies testing the hypothesis that C3aR antagonists reduce disease severity in patients with MN. The present pathophysiologic mechanism may apply not only to MN cases associated with anti-PLA2R and anti-THSD7a autoantibodies but also to other MN cases in which glomerular deposition of autoantibodies activates the complement cascade, further increasing the significance of our findings.

## Methods

A schematic representation of the experimental methodology of this study can be found in Supplemental Figure 14.

### Sex as a biological variable

Our study examined male mice, since female mice are resistant to the development of the disease. Cell lines and human sera used in this work were sourced from both male and female patients, and similar findings are reported for both sexes.

### Cell harvesting, isolation, and expansion

#### Primary cells.

Kidneys deemed nonsuitable for transplantation (from patients with a nonnephrological cause of death) were used for isolation of hPOD and hGEC as previously described (13). hPODs were used within 3 passages from isolation (p0), while hGEC were used within 7 passages from isolation to ensure the correct phenotype. CHLA IRB approved tissue collection. A detailed description of the methodology can be found in our previous publications (13, 15) and Supplemental Methods.

#### hAKPC.

For experiments requiring extensive culture time that could cause hPOD to lose their phenotype, human amniotic fluid–derived podocytes were used (13, 16). Cells were prepared for sorting as described below. A detailed description of the methodology can be found in our previous publications (13, 16) and Supplemental Methods.

### FACS and flow cytometry analysis

hPOD and hGEC were isolated from human glomerular cell suspension by staining with, respectively, NPHS1-FITC (LS-C370063, LSBio [LSBio]) and CD31-AF647 (561654, BD Pharmingen) antibodies (13). Briefly, cells were blocked using 1× human IgG (MilliporeSigma, I2511) for 30 minutes and then stained with the specified antibodies, 1 μg/1 × 10^6^ cells/100 μL IgG solution unless otherwise specified on the data sheet, for 1 hour on ice. Cells were then washed twice in PBS and filtered immediately before sorting. Cells were sorted using a FACSAria sorter (BD Biosciences). Unstained and single-positive controls were used to perform area scaling, exclude autofluorescence, and perform fluorochrome compensation when needed. Cells were first gated based on forward scatter and side scatter (FSC/SSC) to exclude dead cells and then gated for FSC-width/FSC-height (FSC-W/FSC-H) and SSC-W/SSC-H to exclude potential duplets. Sorting gates were established based on the unstained population for each sample. Total amniotic fluid population cells were labeled with anti-CD24 (1 mg/mL, Abcam, ab134375), anti–OB-Cadherin (1 mg/mL, Abcam, ab151302), and anti-podocalyxin (1 mg/mL, Abcam, ab150358) and antibodies marked with Zenon kit fluorochromes (Alexa Fluor 488, Alexa Fluor 647, and R-PE; Invitrogen). Cells were sorted via a FacsAria flow cytometer (BD Biosciences) (16). Sorted cells were expanded in CHANG medium (13, 15). Differentiation of hAKPC was performed by culturing them on collagen I–coated plates in VRADD media for up to 2 weeks (15).

### Immunofluorescence and confocal imaging

Immunofluorescence staining was performed on hPOD, hAKPC, and hGECs: following fixation by 4% paraformaldehyde for 10 minutes at room temperature (Santa Cruz Biotechnology, sc-281692) and serial washes with PBS. The following antibodies were used for the immunostainings: Nephrin (LS-C370063, LSBio), WT1 (ab15249, Abcam), CD31 (561654, BD Pharmingen), EHD3 (HPA049986, Atlas Antibodies), PLA2R (MABC942, MilliporeSigma), C5a9b (NBP1-05120, Novusbio), C3aR (NBP2-15649, NovusBio), IgG4 (LS-C351418-500, LSBio). Chips/wells of interest were prepared for staining by blocking with 5% BSA (Jackson ImmunoResearch, 001-000-162) in PBS for 30 minutes. Primary, secondary, and preconjugated antibodies were diluted in 2.5% BSA (Jackson ImmunoResearch, 001-000-162) as indicated in Table 1. Fluorescence images were captured by a Leica DMI 6000B microscope.

For staining on OrganoPlate, 30 μL of solution were added to the top and bottom inlets and outlets of the chips or 100 μL of the solution was added directly into the chamber slide wells. Primary antibodies were incubated at room temperature (RT) for 1 hour; following serial washes, secondary antibodies were incubated at RT for 30 minutes. After a final series of washes in PBS, DAPI was applied (1:1,000 in PBS; BD Pharmingen, 564907) and the OrganoPlate or the wells were stored at 4°C until imaged by confocal microscopy (Zeiss 710 microscope) and processed using the ZEN11 software (Zeiss, Germany).

### Western blotting

Total cell lysates were prepared using Total Exosome RNA & Protein Isolation Kit (Invitrogen, 4478545), according to manufacturer instructions. Protein concentration was measured with Bradford assay (Thermo Fisher Scientific, 23236). An approximately equal amount of protein was loaded and separated on 4%–15% Criterion TGX Precast Midi Protein Gel (BIO-RAD, 5671083) in at least triplicates for each sample group. Proteins were probed with various antibodies at 1:1,000 concentration overnight at 4°C. The following primary antibodies were used: PLA2R (LS-C108906-100, LSBio), C3AR (NBP2-15649, NovusBio), caspase-3 (9662S, Cell Signaling Technology), Synaptopodin (ab224491, Abcam), ROMO1 (NBP2-45607, NovusBio), C5b-9 (bs-2673R, Bioss), and THSD7A (HPA000923, Atlas Antibodies). Peroxide conjugation of secondary antibodies, goat anti–mouse IgG (H+L) (catalog A28174) or goat anti–rabbit IgG (H+L) (superclonal recombinant secondary antibody, A9169, 2 mL, Sigma-Aldrich) was done for 30 minutes at RT, and the signal was detected for 3–5 minutes using chemiluminescence detection reagent (SuperSignal West Pico Plus, 34577; West Femto Maximum, 34096; Thermo Fisher Scientific) on Biomax Light Film. Image Studio Lite Quantification Software was used to analyze and compare at least 3 replicates on separate Western blots; normalization was done by using an internal loading control, housekeeping protein either β-actin (GeneTex, GTX109639), or GAPDH (Cell Signaling Technology, 971665). Fold change was calculated by the ratio of sample signal density to housekeeping protein signal density.

### qPCR

Total RNA was extracted from fresh or frozen cell samples by Direct-zol RNA miniprep Kits (ZYMO, 2052). RNA quality was determined by analysis of the A260/A280 ratio, and concentration was detected by Nanodrop (Thermo Fisher Scientific NanoDrop 2000). Following reverse transcription (Thermo Fisher Scientific, 4387406), amplification was performed using the SYBR Green quantitative PCR (qPCR) SuperMix (Thermo Fisher Scientific, 1176002K) on the LightCycler 480 System, 96 wells (Roche LightCycler 480). Samples were run in duplicate.

### ELISA

C3a protein levels in the sea and media were quantified with the Human C3a ELISA kit from Invitrogen (catalog BMS2089) according to the manufacturer instructions. Human sera were diluted to 0.5% in culture medium without FBS and added to the GOAC. After 24 hours, the media were collected from the top and bottom channels. Freshly prepared media with 0.5% serum were used as a control. Samples were transferred in duplicates to a 96-well plate precoated with C3a antigen and then incubated at 25°C for 2 hours. The wells were washed 6 times, with washing buffer from the kit, and then coincubated with enzyme-conjugated secondary antibodies at 25°C for 1 hour. A microplate reader (Wallac 1420; Perkin Elmer) was used to measure absorbance at 450 nm. Standard C3a was diluted in serial 2-fold steps, as described in the protocol, and values from the standard curve were used to determine concentrations in our samples.

### Silencing of C3aR and PLA2R

To perform silencing, a shRNA(h) lentiviral particles transduction system was utilized on hAKPC. Undifferentiated hAKPC cells were expanded in CHANG medium (13, 16) to reach 70%–80% confluence, before being transduced at 5MOI (multiplicity of Infection [MOI], the number of lentiviral particles per cell). Cells were cultured in a mixture of complete medium with polybrene (SC-B4220, lot no. K2019, Santa Cruz Biotechnology) at a final concentration of 10 μg/mL. Transduction was performed using Lentiviral particles for C3aR shRNA(h) (Santa Cruz Biotechnology, sc-42840-V, lot no. D1715) and PLA2R shRNA(h) (Santa Cruz Biotechnology, sc-94746-V, lot no. G2911) for 72 hours to knock down C3aR and PLA2R, respectively. Scrambled sequences (Control shRNA Lentiviral Particles-A, sc-108080, Santa Cruz Biotechnology) and empty vector control were used as controls. Selection medium containing puromycin 5 μg/mL (Sigma-Aldrich, SBR00017) was used to remove nontransfected cells for as long as 2 weeks; knockdown efficiency was tested and verified via qPCR using C3aR (Origene, HP207426), PLA2R (Origene, HP234354), and ACTB (Origene, HP204660) primers and Western blotting as previously described. Experiments were approved by CHLA Institutional Biosafety Committee.

### GOAC setup

OrganoPlate culture was performed using a 3-lane chip with 400 μm × 220 μm channels (Mimetas BV, Netherlands) as previously described (13, 16). A detailed description of the methodology can be found in our previous publications (13, 16) and Supplemental Methods.

### Experiments with human sera

Deidentified sera from healthy subjects and individuals with MN (anti-THSD7A, *n* = 5; anti-PLA2R, *n* = 3) were obtained from Coralien Vink and Jack Wetzels (Department of Nephrology, Radboud University Medical Center, Nijmegen, Netherlands). Healthy control sera were obtained by Paolo Cravedi (Icahn School of Medicine at Mount Sinai) and Andrea Angeletti (University of Bologna, Italy). Protocols for the collection of these human samples were approved by the IRBs of these institutions, and informed consent was obtained from all participants.

### Albumin permselectivity assay

An albumin permeability assay was performed as previously described (13, 15). Briefly, media were aspirated from the bottom inlet and outlet to which PBS was added. Then, media from the top inlet and outlet were aspirated. In total, 50 μL albumin-FITC (MilliporeSigma, A9771) was added to the top inlet and outlet. The chips were imaged at 5 and 60 minutes, during which the plates continued to incubate at 37°C. At 60 minutes, media were collected from the bottom inlet and outlet. Absorbance was measured using the Perkin Elmer Victor 3 plate reader using Wallac 1420 workstation software (fluorescein 485/535, 0.1 seconds). Albumin leakage was quantified as the fluorescence signal of the filtrate collected from Channel F.

### IgG neutralization

IgG neutralization was performed by the addition of IdeS (18), used at a concentration of 20 ng/μL of serum, to the healthy or MN sera prior to exposure to the GOAC at an adjusted concentration of 0.5% (accounting for the dilution due to the IdeS) for 24 hours. Albumin leakage readouts were performed as previously described.

### Complement inactivation

Complement inactivation was attained by heating the serum to 56°C for 30 minutes before exposure to the chip. The heat-inactivated sera were added to the chip at a concentration of 0.5% for 24 hours as previously described. Albumin leakage readouts were performed as previously described.

### C5b-9 inhibition

Inhibition of MAC formation was obtained by the addition of 50μg/mL human protein S (19, 20) (Enzyme Research Laboratory #HPS) to healthy and MN sera, prior to the exposure to the chip at a concentration of 0.5% for 24 hours as previously described. Efficiency of MAC inhibition was confirmed by immunofluorescence and confocal microscopy as described above. Additionally, exposure of a GOAC to C6-deficient human serum (C1288, Sigma-Aldrich) was assessed to further confirm the role of MAC. Albumin leakage readouts were performed as previously described.

### Exposure of GOAC to recombinant C3a, C5a, C3aR, and C5aR antagonist

We exposed GOAC to 50 nM C3a (3677-C3-025, R&D) or C3a + C3aR antagonist (C3aRA, SB290157, Cayman Chemical) in GOAC culture media for 24 hours as previously described (13, 15, 21). In a complementary experiment, we exposed GOAC to 50 ng/mL C5a (R&D, 2037-C5), C5a + 25 μg/mL C5aR antagonist (Sigma-Aldrich, 533683), or MN + C5aR antagonist. Albumin leakage readouts were performed as previously described (13, 15).

### Animal experiments

Sera obtained from patients with THSD7A-associated MN were used for disease induction (Table 1). The protocol for disease induction was modified from Tomas et al. (14). Prior to injection, human sera were concentrated using Amicon Ultra-15 centrifugal filters with a molecular cut-off of 100 kDa (EMD Millipore), sera were decomplemented by heating for 30 minutes at 56°C in a water bath, and the remaining debris was removed by centrifugation at 20,000*g* for 15 minutes at RT. BALB/c background mice (The Jackson Laboratory) of at least 20 g and 8 weeks of age were injected i.p. with 200 μL of adjusted sera once for disease induction. We backcrossed the B6 *DAF*^–/–^ animals 14 generations to BALB/c to produce BALB/c *DAF*^–/–^ animals (21) since we have previously shown that lack of DAF enhances complement-mediated injury in vivo (21). Free water access and a normal diet were maintained over the course of the study.

Development of albuminuria was monitored by weekly urine collection from individual mice before treatment and at weekly intervals until sacrifice. Urinary creatinine was quantified using commercial kits (Cayman Chemical). Urinary albumin was determined using a commercial assay from Bethyl Laboratories (E99-134). Urinary albumin excretion was expressed as the ratio of urinary albumin/creatinine. Mice were sacrificed at 1 or 8 weeks after serum injection.

C3aRA (SB290157; Cayman Chemical) or vehicle control was administered through i.p. injection beginning the day prior to disease induction at the dose of 10 mg/kg biweekly (powder dissolved in PBS and 10% DMSO) until euthanasia.

### Renal histology

The pathogenicity of anti-THSD7A antibodies in mice was verified through histological analysis. Mice were anesthetized with a 100 μL i.p. injection of a solution made of sterile ketamine (16 mg/mL) and xylazine (7 mg/mL) in Gibco PBS (Thermo Fisher Scientific) and transcardially perfused with periodate-lysin-paraformaldehyde fixative at 4% in PBS. Kidneys were harvested and frozen in optimal cutting temperature compound (Tissue-Tek O.C.T.; Sakura Finetek) or embedded in paraffin. Paraffin-embedded kidney sections (3 μm) were stained with periodic acid–Schiff (PAS) or Jones’ stain (methenamine silver-PAS stain).

### Immunofluorescence on mouse tissue

For mouse kidney samples, Tissue-Tek O.C.T. preserved cryosections (5 μm thick) were washed with PBS for 15 minutes and then left for 60 minutes at room temperature with blocking solution (2% BSA, 2% FBS, and 0.2% fish gelatin in PBS). AffiniPure Fab fragment goat anti–mouse IgG (H+L, 115-007-003, Jackson ImmunoResearch) was subsequently applied for 3 hours, followed by incubation at 4°C overnight or room temperature for 1 hour with specific primary antibodies. Sections were then washed and incubated with the appropriate secondary antibody for 60 minutes at room temperature: anti–mouse IgG antibody conjugated with Alexa Fluor 594 (1:200; Thermo Fisher Scientific), anti–hamster IgG antibody conjugated with Alexa Fluor 488 (1:200; A21110, Thermo Fisher Scientific), anti–rat IgG antibody conjugated with Alexa Fluor 568 (1:500; A-78946, Invitrogen), anti–rabbit IgG antibody conjugated with Alexa Fluor 594 (1:500; A-11077, Invitrogen), anti–mouse IgG antibody conjugated with Alexa Fluor 594 (1:500; 115-585-003, Jackson ImmunoResearch), and anti–goat IgG antibody conjugated with Alexa Fluor 594 (1:500; A-11012, Invitrogen). Nuclei were counterstained with DAPI mounting media (ProLong Gold antifade reagent with DAPI, P36931; Invitrogen). Antibody expression was estimated by constructing a contour mask on the bright-field image. ImageJ software (NIH) was used to quantify staining intensity.

### Statistics

Unless indicated, data are shown as the mean ± SEM. Statistical significance was calculated using 1-way ANOVA, 2-way ANOVA, Mann-Whitney *U*, Kruskal-Wallis test, and 2-tailed Student *t* test. Where appropriate, *P* values have been corrected for multiple comparisons. *P* values of less than 0.05 were considered significant.

### Study approval

Silencing experiments in vitro were approved by the Institutional Biosafety Committee at CHLA (protocol no. IBC-19-018). Collection of clinical serum samples were approved by the IRB at the University of Bologna (BO-PBMC 420/2018/Oss/AOUBo) and Radboud University Medical Center; informed consent was obtained from all participants. Animal study protocols were approved by the IACUC at Icahn School of Medicine at Mount Sinai (New York, New York, USA; IACUC ID PROTO202000090). The reference no. of the OLAW-approved Animal Welfare Assurance of Icahn School of Medicine at Mount Sinai is D16-00069 (A3111-01).

### Data availability

All data needed to evaluate the conclusions in the paper are present in the paper or the Supporting Data Values file. Any additional information required to reanalyze the data reported in this paper is available upon request.

## Author contributions

QZ and SB share the first authorship based on their overall contribution to the current manuscript in terms of data generation, analysis, and manuscript revision. QZ is listed first due to her contribution to project conceptualization. QZ, KB, AP, and VV conceptualized the study. QZ, SB, KB, AP, VV, PA, PM, HS, SS, and SDS acquired and analyzed data. QZ, SB, KB, LP, PC, and SDS interpreted data. CV, JW, and PC collected and provided human clinical samples. GLM, PC, LP, KVL, REDF, PC, and SDS supervised and mentored the staff and contributed to study design. PC and SDS supervised the study. PC and SDS acquired funding. QZ, MAP, LP, PC, and SDS wrote the original draft of the manuscript. All authors read, edited, and approved the final manuscript.

## Supplementary Material

Supplemental data

Supporting data values

Unedited blot and gel images

## Figures and Tables

**Table 1:**
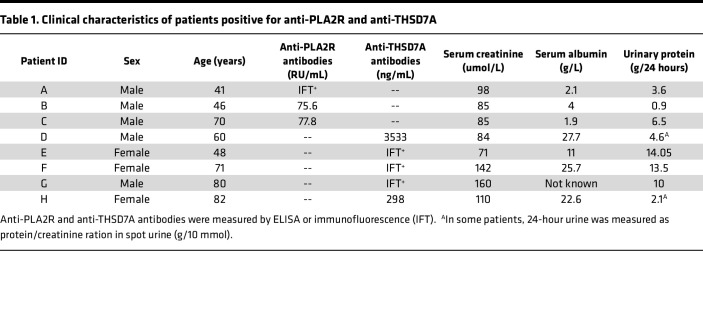
Clinical characteristics of patients positive for anti-PLA2R and anti-THSD7A

## Version 1 01/16/2024

In-Press Preview

## Version 2 02/22/2024

Electronic publication

## Version 3 03/27/2024

Updated the affiliations for Drs. Perrin and Da Sacco
