# Supplementary material for: C3aR-initiated signaling is a critical mechanism of podocyte injury in membranous nephropathy
Source: JCI Insight. 2024 Jan 16;9(4):e172976. doi: 10.1172/jci.insight.172976 (PMC11143932; doi:10.1172/jci.insight.172976)
Supplement: Supplemental data [file jciinsight-9-172976-s069.pdf]

Supplementary Figures

Figure S1: Characterization of primary podocytes and GEC

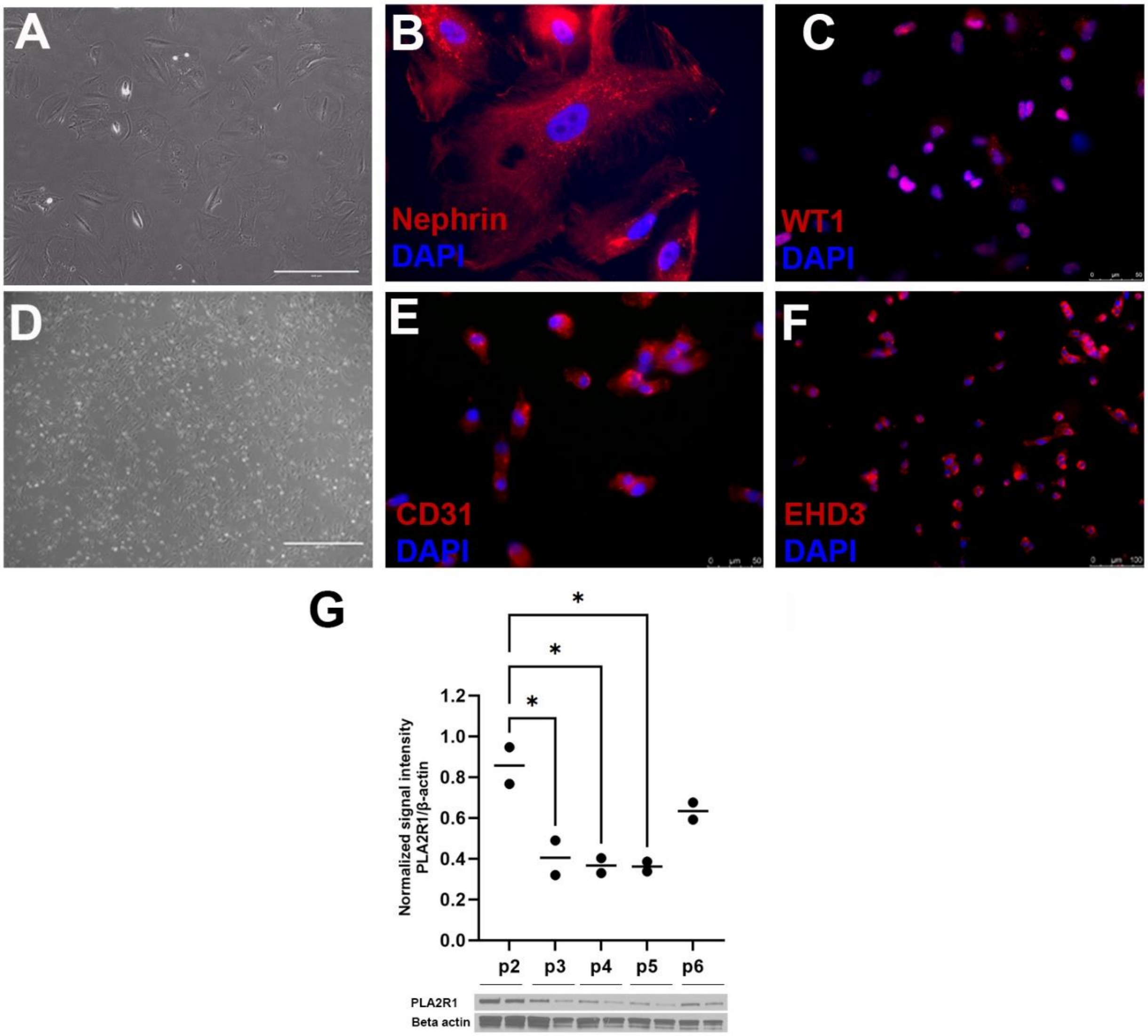

A: Morphology of primary podocytes after selection for nephrin. Scale bar: 400µm. **B-C**: Immunofluorescence staining for nephrin (red, **B**, Scale bar: 25µm) and WT1 (red, **C**, Scale bar: 50µm) confirming the maintenance of podocyte phenotype upon isolation. DAPI nuclear staining: blue. **D:** Morphology of human glomerular endothelial cells (GEC) after selection for CD31. Scale bar: 1000µm. **E-F:** Immunofluorescence staining for CD31 (red, **E**, Scale bar: 50µm) and EHD3 (red, **F**, Scale bar: 100µm) confirming

the maintenance of GEC phenotype upon isolation. DAPI nuclear staining: blue. **G.** Western blotting analysis of PLA2R1 in hPOD at different passages. Measured density for PLA2R1 bands were normalized against beta actin, showing no significant change at the protein level. Number of replicates/group: 2; PLA2R1: 150 kDa; beta actin: 42 kDa; Positive control: see additional supplementary file for full unedited membrane that includes the positive control band for recombinant PLA2R1 protein (#TP313576, Origene): 154 kDa. significance * $p<0.05$. All statistical values determined by One Way ANOVA.

Figure S2: Effect of anti-PLA2R serum on albumin leakage on the GOAC

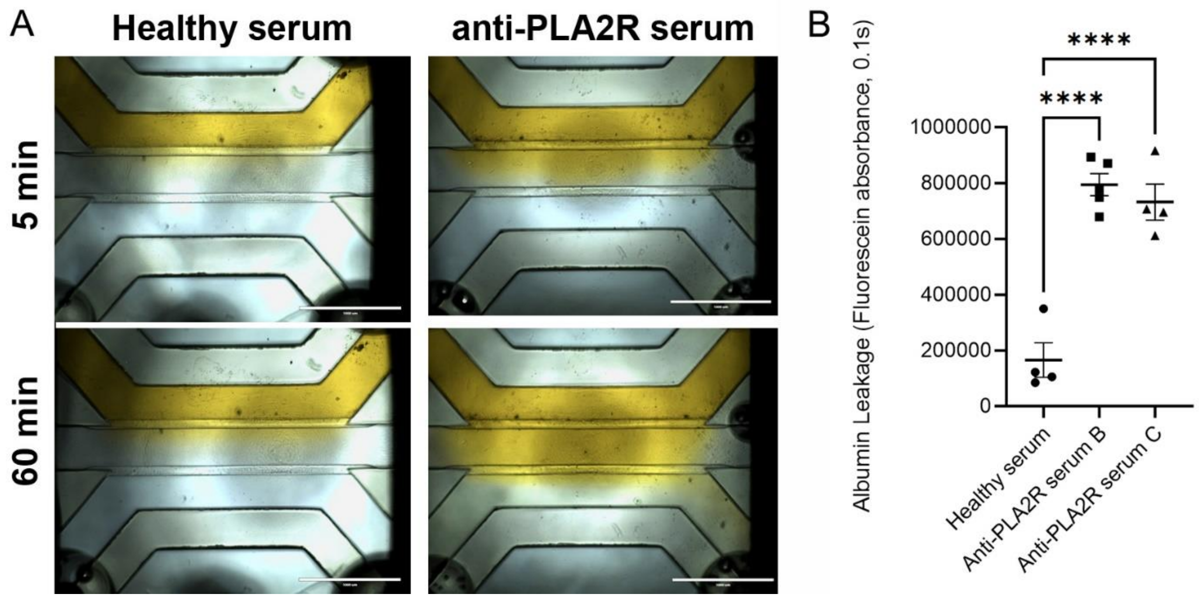

A. Representative bright field pictures showing albumin leakage (yellow, central panels) after 5 min (top row) and 60 min (bottom row) in GOAC exposed to healthy serum or anti-PLA2R serum. Marked albumin leakage occurs following podocyte injury by anti-PLA2R serum. **B.** box plot graph of fluorescein absorbance (485nm/535nm, 0.1s) in filtrate collected from channel F 60 minutes after addition of albumin, following exposure to anti-PLA2R MN patient serum at a concentration of 0.5% for 24 hrs. A statistically significant increase in albumin leakage was confirmed in chips exposed to MN sera B and C compared to those exposed to healthy individual sera (significance ****p<0.0001, n of technical replicates≥4/group). All statistical values determined by One Way ANOVA.

Figure S3: C3 and C5b-9 expression in GOAC exposed to MN serum

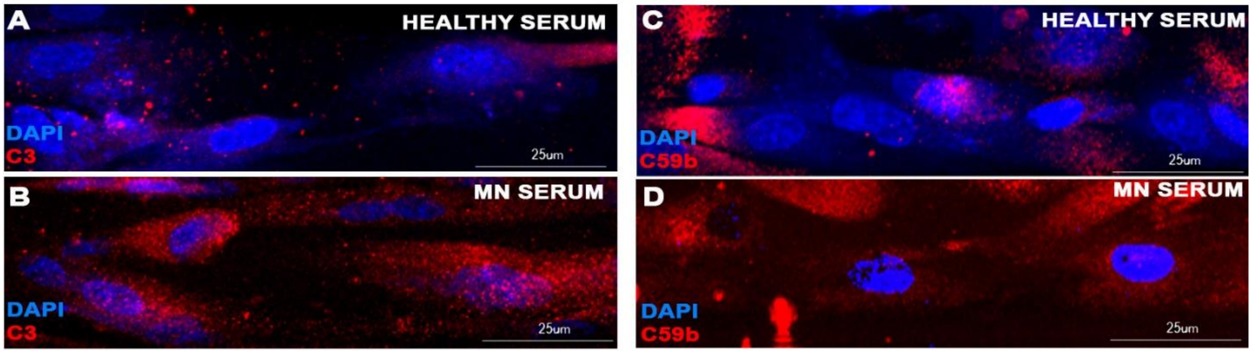

A-D. Representative images of confocal microscopy of staining for C3b (A-B) and C5b-9 (C-D) on GOAC following exposure to healthy serum (A, C) or MN serum (B, D). GOAC exposed to MN serum shows increased C3b and C5b-9 deposition compared to GOAC exposed to healthy control serum. C3b and C5b-9 were stained in red, Nuclei were stained with DAPI in blue. Scale bar: 25 μm

Figure S4: Effect in the GOAC of PLA2R1 silencing on GEC

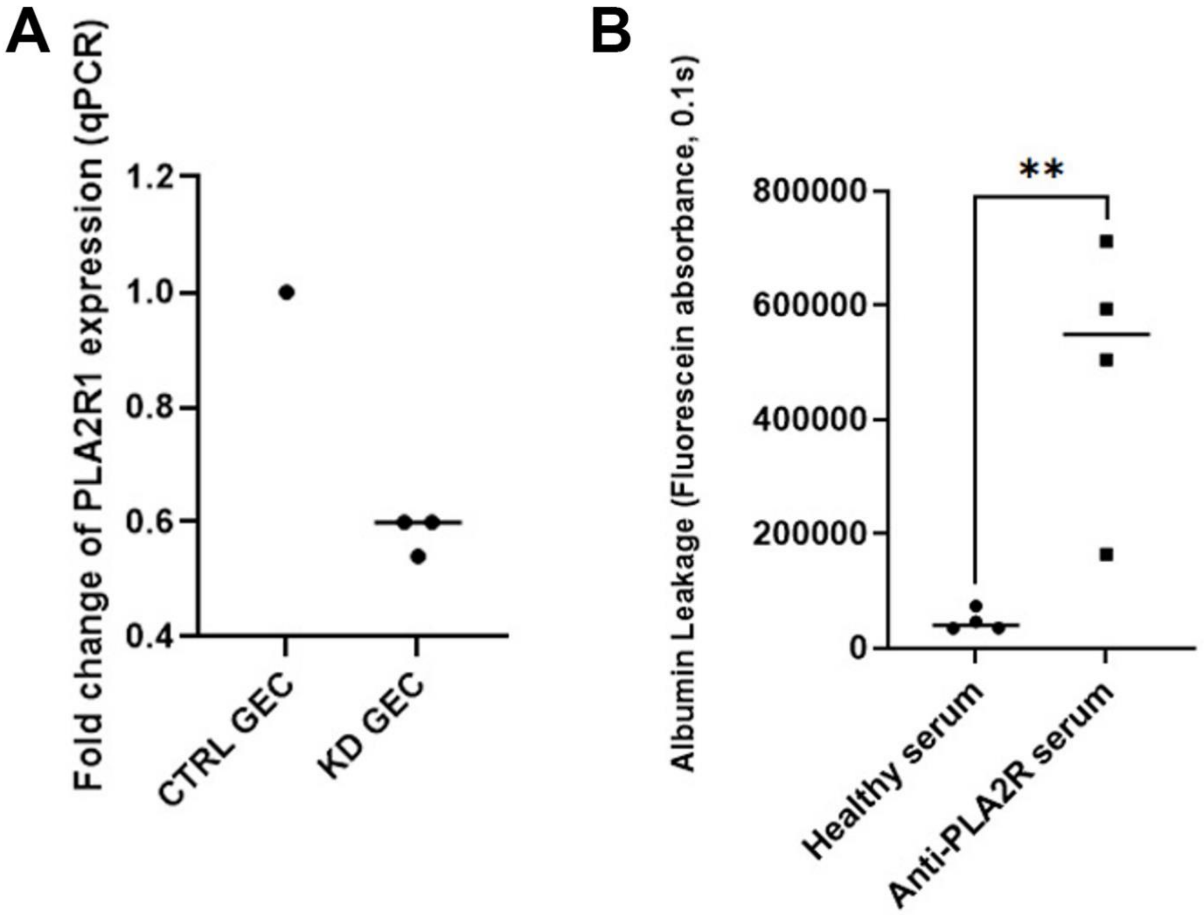

A. Box plot graph showing decrease in PLA2R1 RNA expression in GEC following stable silencing. Control value, obtained from the average of 3 unmodified GEC replicates, arbitrarily assigned to 1 to calculate fold change. A reduction of about 40% in PLA2R expression was confirmed. **B.** Box plot graph of fluorescein absorbance (485nm/535nm, 0.1s) in filtrate collected from channel F 60 minutes post albumin addition. Exposure of GOAC to 0.5% anti-PLA2R+ serum for 24hrs in the group generated with GEC KD for PLA2R1 increased albumin leakage compared to the control group; Silencing of PLA2R1 in GEC is unable to prevent albumin leakage on the GOAC (significance ** $p<0.01$; n of replicates≥4/group). All statistical values determined by Student's *t*-test.

Figure S5: Immunofluorescence staining of IgG deposition

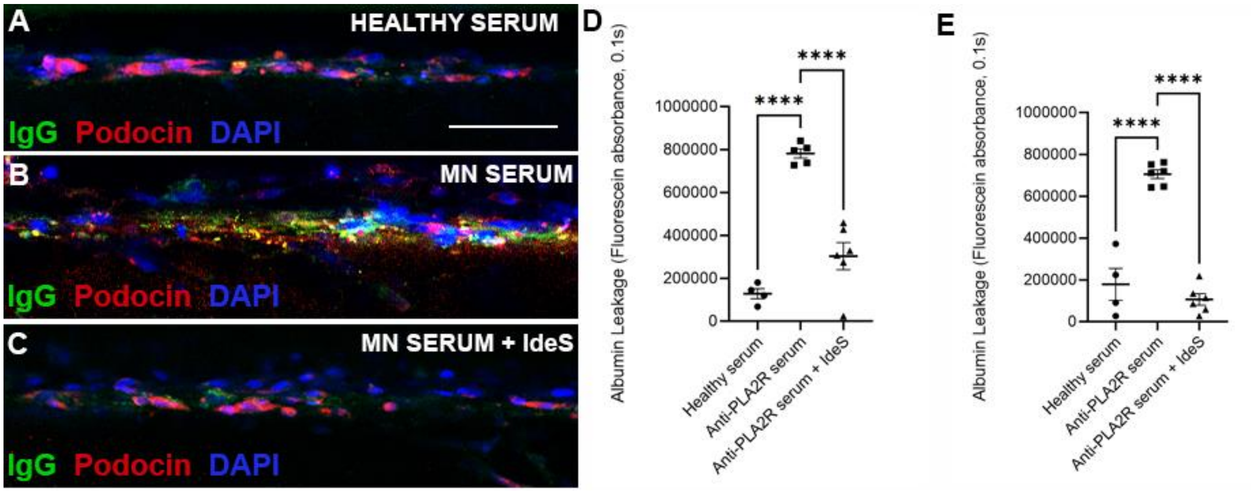

A-C. Representative images of confocal microscopy of staining for total IgG deposition (green) on podocytes (red, stained for podocin) in the GOAC following exposure to healthy serum (no deposition) or MN serum with or without IdES in which IgG deposition is clearly detectable in the MN serum group (**B**) but not in the MN serum + IdeS cohort (**C**). Nuclei were stained with DAPI in blue. Scale bar: 100 μm. **D-E.** Box plot graph of fluorescein absorbance (485nm/535nm, 0.1s) in filtrate collected from channel F 60 minutes after addition of albumin. Enzymatic neutralization of antibodies by the IdeS system in MN sera B (**D**) and C (**E**) prevents leakage on the GOAC. (significance, ****p<0.0001, n of replicates≥4/group). All statistical values determined by One Way ANOVA.

Figure S6: Effect of heat-inactivation on albumin leakage on the GOAC

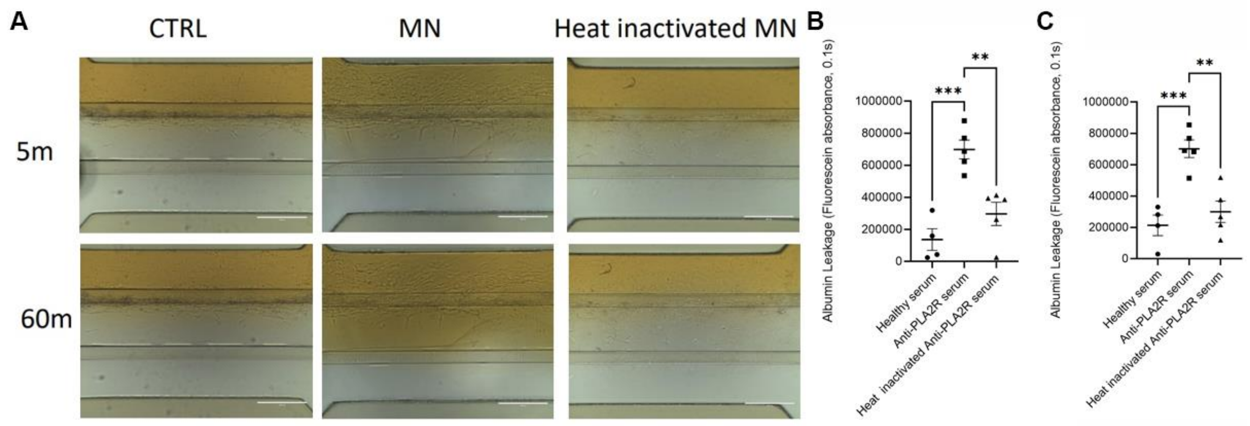

A. Representative bright field pictures showing albumin leakage (yellow, central panels) after 5 min (top row) and 60 min (bottom row) in GOAC exposed to healthy serum, MN serum or heat-inactivated MN serum. Marked albumin leakage occurs following podocyte injury by MN serum but is prevented by complement inactivation. **B-C.** Box plot graph of fluorescein absorbance (485nm/535nm, 0.1s) in filtrate collected from channel F 60 minutes after addition of albumin. Complement neutralization by heat-inactivation successfully prevents injury on GOAC caused by MN patient sera B (**B**) and C (**C**). (significance ** $p<0.01$, ***$p<0.001$, n of replicates≥4/group). All statistical values determined by One Way ANOVA.

Figure S7: Expression of C3aR in podocytes upon culture

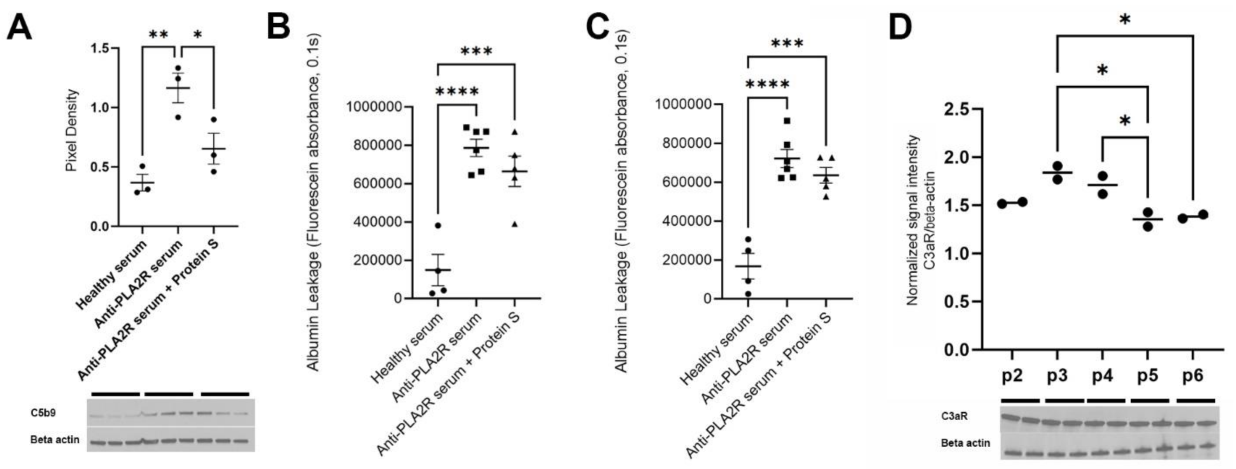

A. Western blotting analysis of C5b9 in hPOD exposed to healthy serum or anti-PLA2R serum with or without Protein S. Measured density for C5b9 bands were normalized against beta actin, showing an increase in the anti-PLA2R cohort that was prevented by protein S. Number of replicates/group: 3; C5b9: 70 kDa; beta actin: 42 kDa; significance * p <0.05; ** p <0.01. **B-C.** Box plot graph of fluorescein absorbance (485nm/535nm, 0.1s) in filtrate collected from channel F 60 minutes after addition of albumin confirming that MAC neutralization in anti-PLA2R sera B (**B**) and C C), does not prevent leakage, suggesting the possibility of an alternative injury mechanism (significance *** p<0.001, **** p<0.0001; n of replicates=4/group). **D.** Western blotting analysis of C3aR in hPOD at different passages. Measured density for C3aR bands werewere normalized against beta actin, showing limited changes at the protein level across passages. Number of replicates/group: 2; C3aR: 54 kDa; beta actin: 42 kDa; Positive control: see additional supplementary file for full unedited membrane that includes the positive control band for recombinant C3aR protein (#TP304730, Origene): 54 kDa. significance * p <0.05. All statistical values determined by One Way ANOVA.

Figure S8: Production of C3a within the GOAC and efficacy of C3aRA in preventing albumin leakage

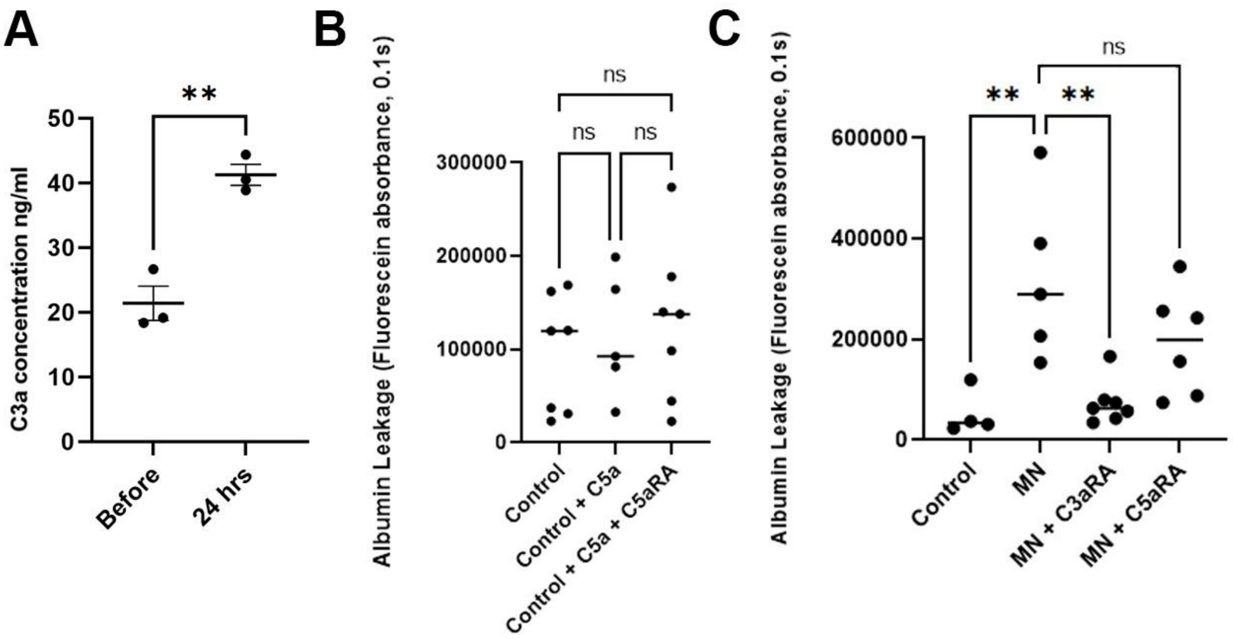

A. ELISA measurements of C3a concentration (ng/ml) in media supplemented with 0.5% anti-PLA2R serum before and after exposure to the GOAC. Levels of C3a increase in 24 hours, suggesting production by cells within the GOAC. When adjusted for the dilution factor, levels of C3a measured in the MN sera used in this experiment range from 3840-5340 ng/ml, consistent with previous findings by Gao et al. Number of biological replicates: 3, significance **p <0.01 **B.** Box plot graph of fluorescein absorbance (485nm/535nm, 0.1s) in filtrate collected from channel F 60 minutes post albumin addition. Exposure of GOAC to 50ng/ml C5a or 50ng/ml C5a + 25ug/ml C5aR antagonist for 24hrs did not cause albumin leakage compared to the control group; (no significance: ns. n of replicates≥5/group). **C.** Box plot graph of fluorescein absorbance (485nm/535nm, 0.1s) in filtrate collected from channel F 60 minutes post albumin addition. Exposure of GOAC to 0.5% anti-PLA2R+ serum for 24hrs increased albumin leakage compared to the control group; Exposure to the chip to 25ug/ml C3aR antagonist

was able to prevent damage, effect not seen by using 25ug/ml C5aR antagonist (significance **$p<0.01$, no significance: ns; n of replicates≥4/group). All statistical values determined by One Way ANOVA with the exclusion of panel A (Student's *t*-test).

Figure S9: Effect of C3aRA on podocytes in the absence of MN serum exposure

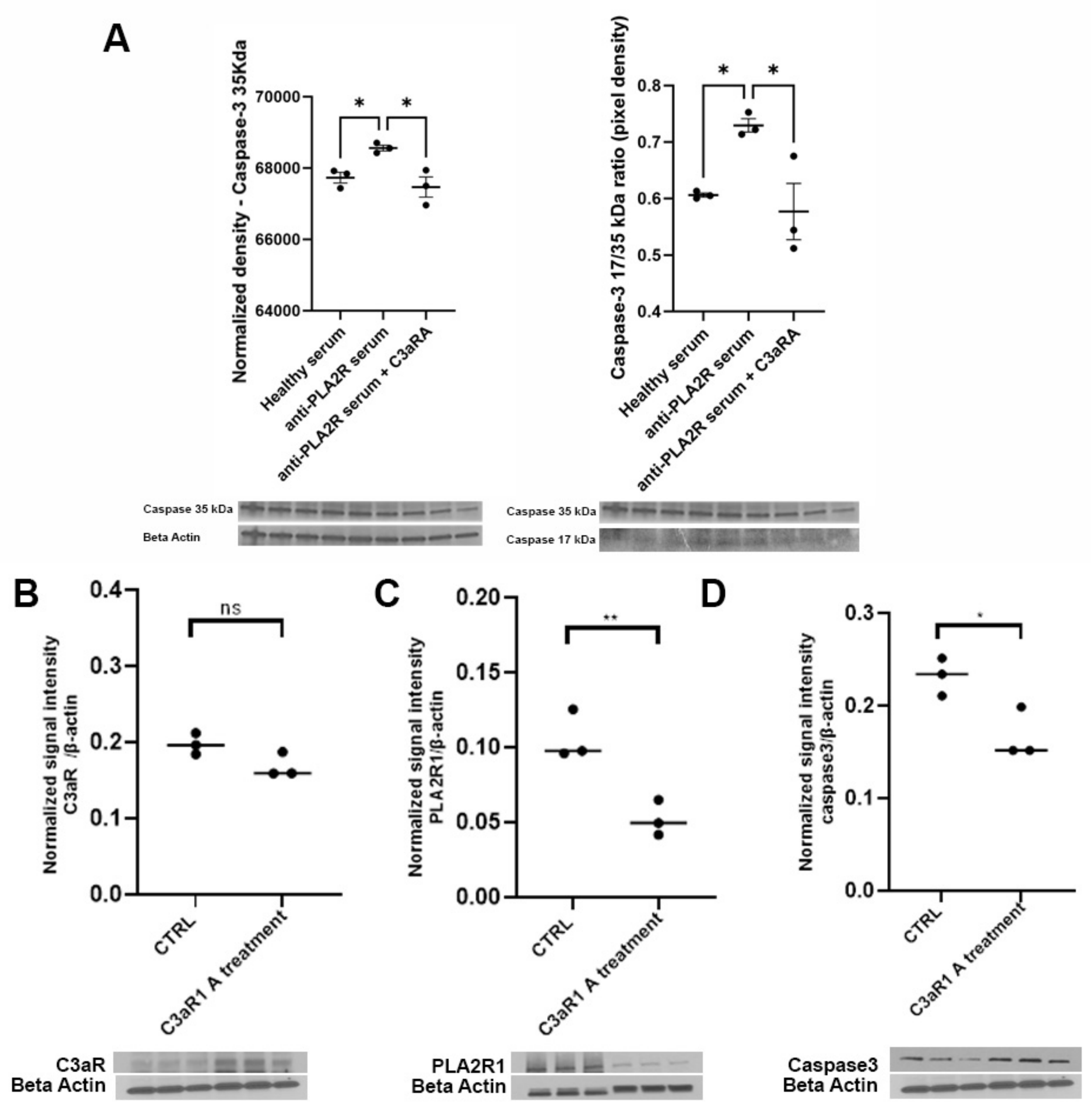

A. Western blotting analysis of apoptosis, measured by expression of caspase 3, in hPOD monolayers exposed to MN sera or MN sera+ C3aRA. The antagonist significantly decreased apoptosis in hPOD. Measured density for Caspase 3 bands were normalized against beta actin (left panel), showing a significantly decreased protein level for uncleaved caspase 3. When ratio between cleaved (17kDa) and uncleaved (35kDa)

caspase 3 was measured, we confirmed that there was a limited but significant increase in apoptosis that was prevented by the C3aRA (right panel) (significance *$p<0.05$;). Number of replicates/group: 3. Caspase3 uncleaved: 35 kDa; beta actin: 42 kDa **B.** Western blotting analysis of C3aR in hPOD exposed to C3aRA, confirming lack of changes after 72 hours. Measured density for C3aR bands were normalized against beta actin, showing no significant change at the protein level. Number of replicates/group: 3 C3aR: 54 kDa; beta actin: 42 kDa **C.** Western blotting analysis of PLA2R in hPOD exposed to C3aRA, showing a statistically significant decrease in protein expression at 72 hours. Measured density for PLA2R bands were normalized against beta actin, showing a significantly decreased protein level. (significance ** $p<0.01$). Number of replicates/group: 3. PLA2R: 150 kDa; beta actin: 42 kDa **D.** Western blotting analysis of apoptosis, measured by expression of Caspase 3, in hPOD exposed to C3aRA. Measured density for Caspase 3 bands were normalized against beta actin, showing a significantly decreased protein level. (significance * $p<0.05$). Number of replicates/group: 3. Caspase3: 35 kDa; beta actin: 42 kDa. All statistical values determined by Student's *t*-test with the exclusion of panel E (One Way ANOVA).

Figure S10: Expression of THSD7A by human primary podocytes

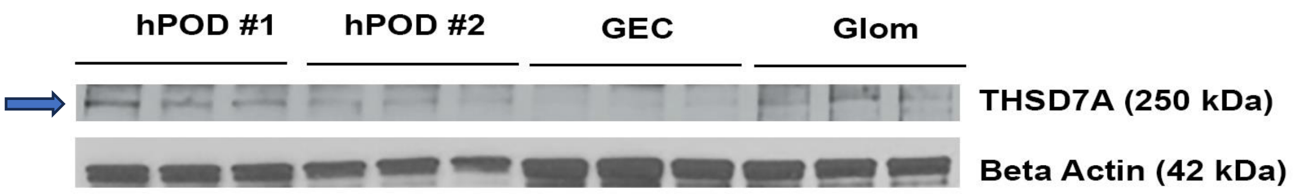

Western blotting analysis of THSD7A in hPOD (two lines), glomerular endothelial cells (GEC), and whole glomeruli confirming expression in podocyte lines (arrow) and glomeruli lysate but not in glomerular endothelial cells alone. Number of replicates/group: 3. THSD7A: 250 kDa; beta actin: 42 kDa

Figure S11: In vivo deposition of human and mouse IgG in mice injected with anti-THSD7A sera

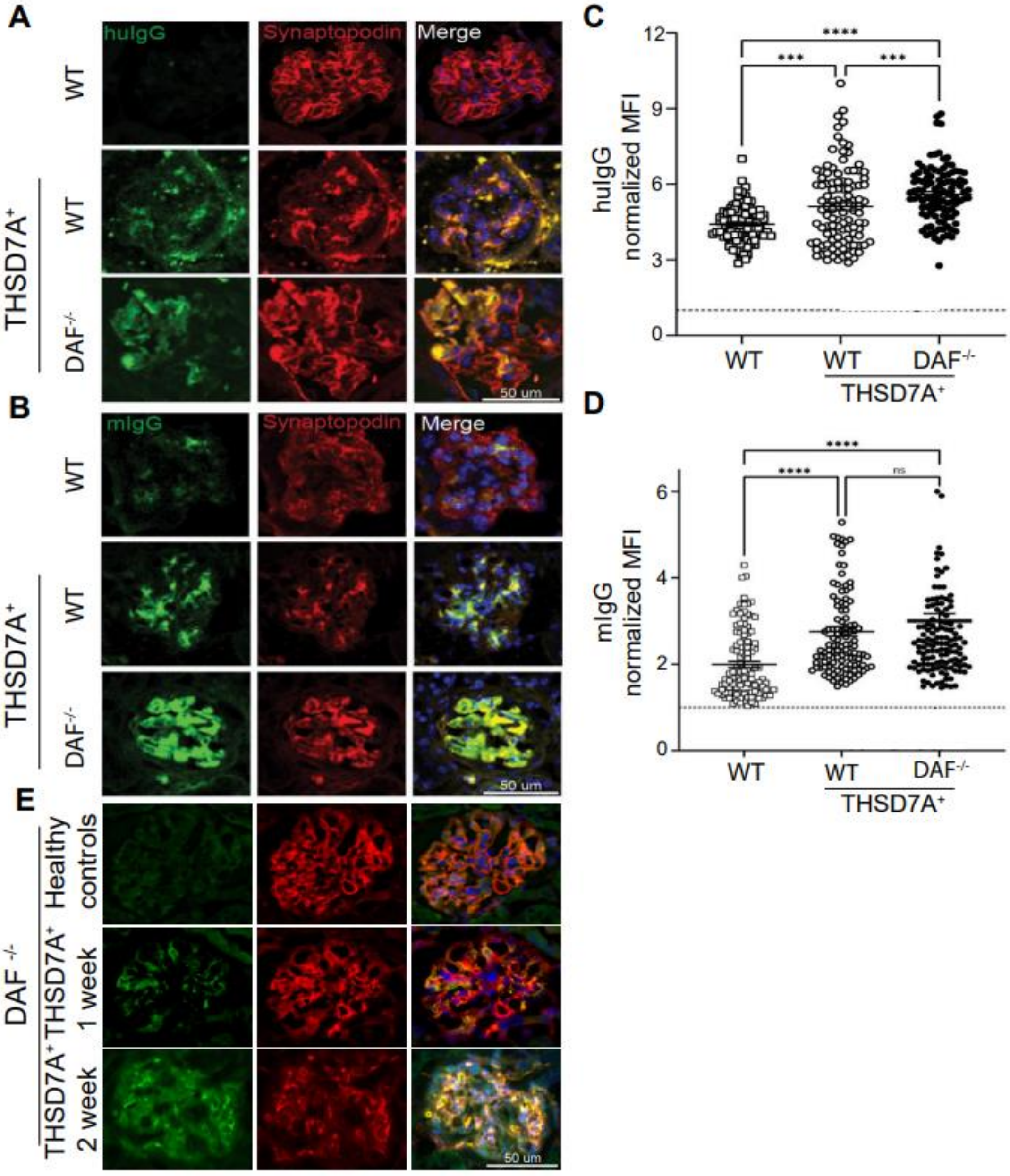

A-B. Representative plots of human (A) and murine (B) IgG glomerular deposition in male WT or DAF$^{-/-}$ mice injected with heat-inactivated serum from patients with membranous nephropathy and anti-THSD7A antibodies. Mice were sacrificed at 8 weeks after serum injection. WT mice injected with heat-inactivated serum from healthy controls were used

as controls. **C-D.** MFI quantification of human (C) and murine (D) IgG glomerular deposition in the same mice represented in A-B. Data refers to 3 mice per group. **E.** Representative plots of murine IgG glomerular deposition at 1 and 2 weeks after injection in DAF$^{-/-}$ mice injected with heat-inactivated serum from healthy controls (top panels) or patients with membranous nephropathy and anti-THSD7A antibodies (lower panels) significance *** $p<0.001$; ****$p<0.0001$. All statistical values determined by Kruskal-Wallis test.

Figure S12: albuminuria in WT mice injection with anti-THSD7A sera

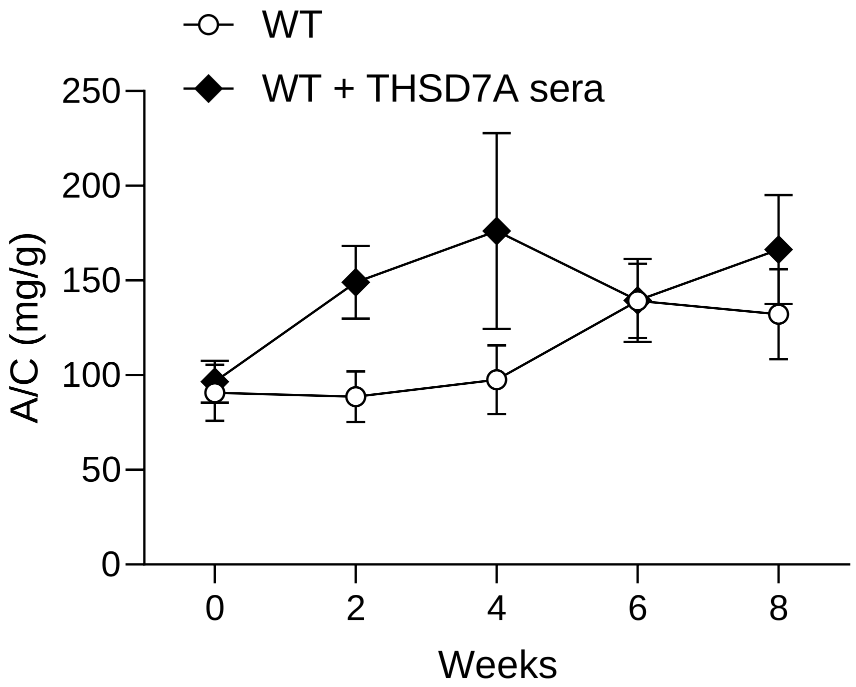

Urinary albumin/creatinine (A/C) levels in WT BALB/c male mice injected with serum from MN patients with anti-THSD7a antibodies. All statistical values determined by Two-way ANOVA.

Figure S13. ***DAF*** **gene expression is increased in glomeruli from MN patients.**

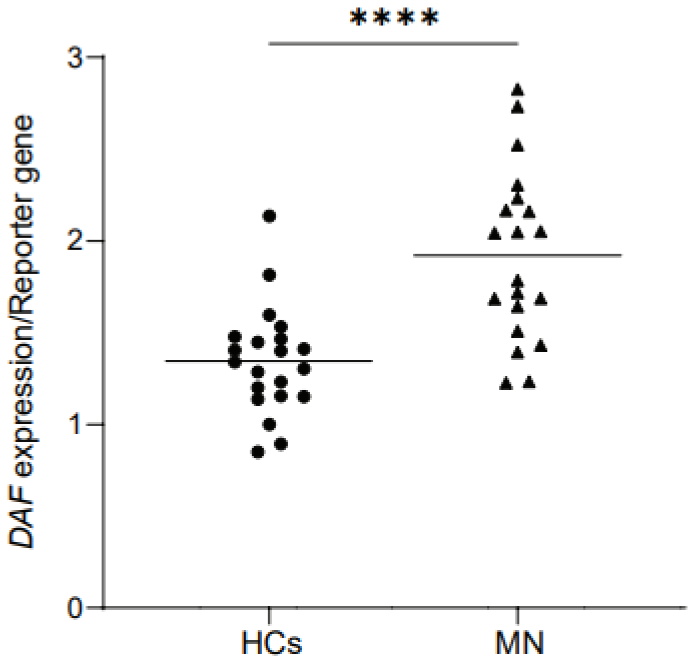

DAF mRNA expression in glomeruli of human biopsy specimens with pathological diagnosis of MN compared with normal kidneys. Data are from previously published microarray studies by Ju et al., 2013 (PMID: 23950145) using Nephroseq (http://www.nephroseq.org/; Accessed on September 15, 2023, significance **** $p < 0.0001$).). All statistical values determined by Mann-Whitney test.

Figure S14: Schematic representation of the experimental methodology

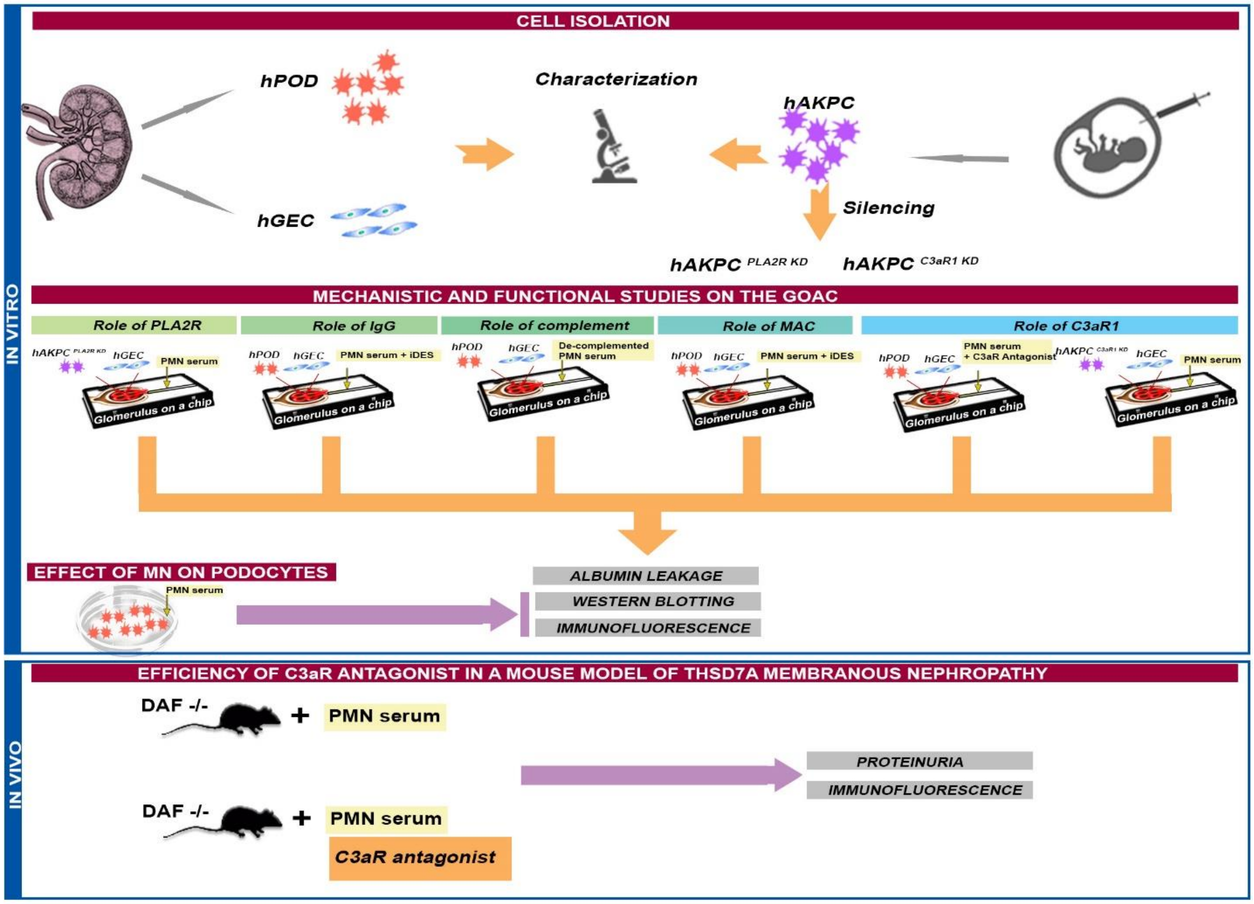

Supplementary Methods

GOAC Setup. 3.0 µl of gel composed of 4 mg/ml Collagen I (AMSbio Cultrex 3D Collagen I Rat Tail, 5 mg/ml, c#3447-020-01), 100 mM HEPES (Life Technologies, c#15630–122) and 3.7 mg/ml NaHCO3 (Sigma, c# S5761) was dispensed in the gel inlet (middle) and incubated 20-30 min at 37°C. hAKPC-P were trypsinized by using 0.05% trypsin-EDTA (Gibco,c#LS25300062), and pelleted by centrifugation at 300g for 5 mins. The cells were applied to the system by seeding 2 µl of 1.5×10^7 of cells/ml in the inlet of the channel C. Subsequently, the OrganoPlate™ was placed on its side at an angle for 30 min at 37°C to allow the cells to sediment against the collagen I. This was followed by the addition of 50 µl of podocyte differentiation medium to both the inlet and outlet of the top medium channel and the OrganoPlate™ was again incubated on its side overnight at 37°C to complete the cell attachment. The following day, GEC were applied to the system using the same procedure as described above. Endothelial cell media was added to the top channel while podocyte media was still used for the lower channels. This created the polarity of the glomerular filtration barrier, with endothelial cells oriented toward the vascular channel represented by our plate, and podocytes oriented toward the urinary channel, which had by then layered on top of the collagen. The OrganoPlate™ was placed horizontally in the incubator (37°C, 5% CO2) on an interval rocker switching inclination every 10 min, allowing bi-directional flow that enables media recirculation within each chip. The media described above was changed every 2-3 days such that endothelial cell medium was added to the top inlet and outlet, and podocyte differentiation medium was added to the bottom inlet and outlet, thereby reaching their respective cell types.
