# Supplementary material for: C3aR-initiated signaling is a critical mechanism of podocyte injury in membranous nephropathy
Source: JCI Insight. 2024 Jan 16;9(4):e172976. doi: 10.1172/jci.insight.172976 (PMC11143932; doi:10.1172/jci.insight.172976)

Full unedited Western Blotting gel images

Full unedited Western Blotting gel image for figure 1G. Original blot images for PLA2R1 and beta actin on control and silenced hAKPC corresponding to blots in Figure 1. PLA2R was measured around 150 kDa; Beta actin at 42kDa. Order of bands (groups of three): Control Whole Kidney Tissue; Scramble; PLA2R1 KD (line used for this Manuscript); Control: whole glomeruli;

PLA2R1

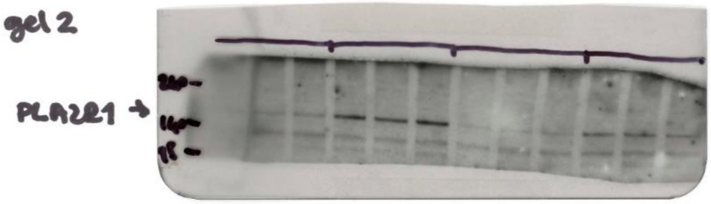

Beta Actin

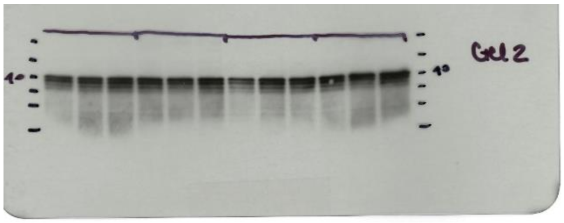

Full unedited Western Blotting gel image for figure 2E. Original blot images for C3aR1 and beta actin on control and silenced hAKPC corresponding to blots in Figure 2E. C3aR1 was measured around 54 kDa; Beta actin at 42kDa. left three bands: scramble; right three bands: C3aR1-silenced hAKPC ;

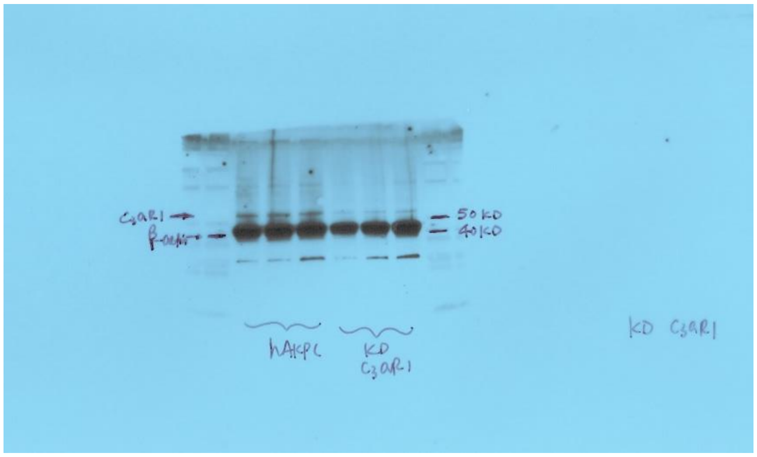

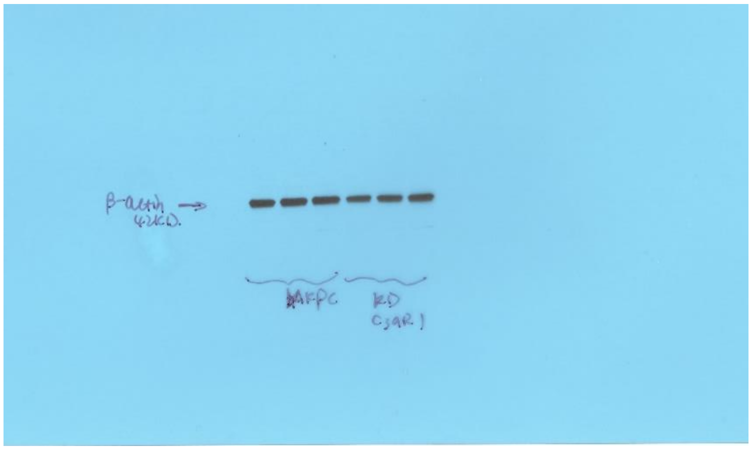

Full unedited Western Blotting gel image for figure 3C. **Original blot images for PLA2R, C3aR1 and beta actin on hPOD exposed to healthy or MN serum corresponding to blots in Figure 3B and 3D. PLA2R was measured around 150 kDa; C3aR1 was measured around 54 kDa; Beta actin at 42kDa.**

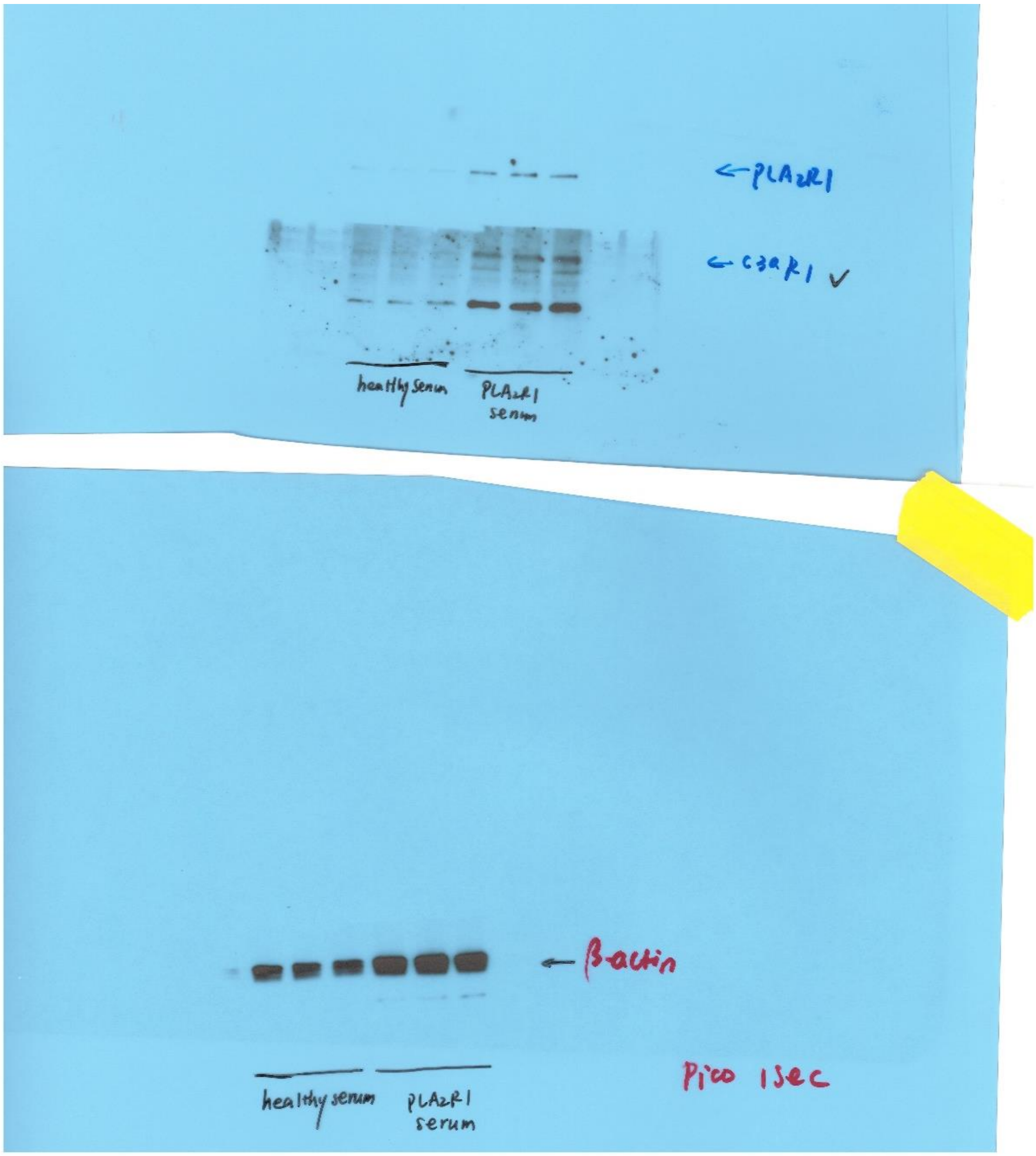

Full unedited Western Blotting gel image for figure 3F. Original blot images for ROMO1, Caspase3 and beta actin on hPOD exposed to healthy or MN serum corresponding to blots in Figure 3E-G. ROMO1 was measured around 10 kDa; Caspase 3 was measured around 19kDa; PLA2R was measured around 150 kDa; C3aR1 was measured around 54 kDa; Beta actin at 42kDa.

First 3 bands: Control (healthy serum); Central 3 bands: anti-PLA2R serum; last 3 bands: not included in current analysis. Endo not included in the current analysis.

Romo1

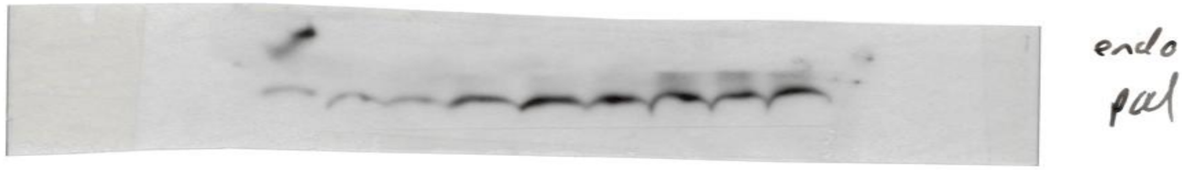

Caspase 3

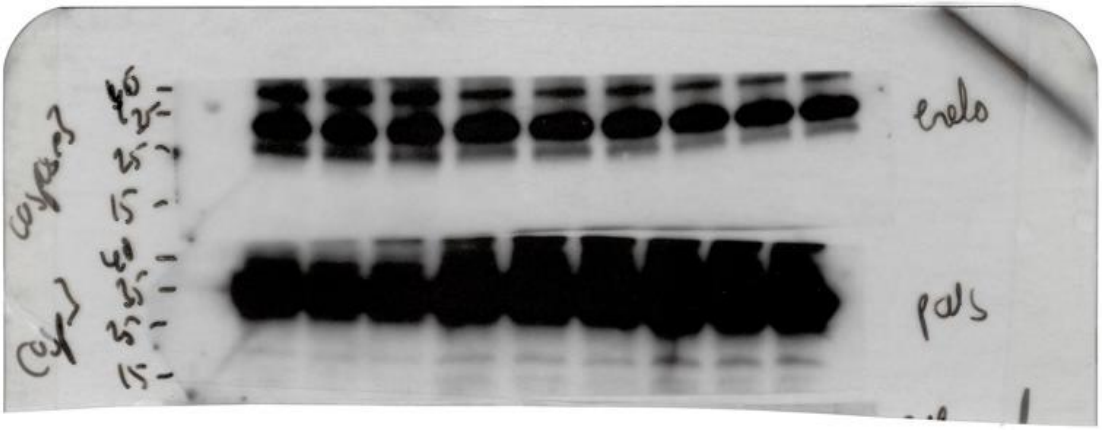

Beta actin

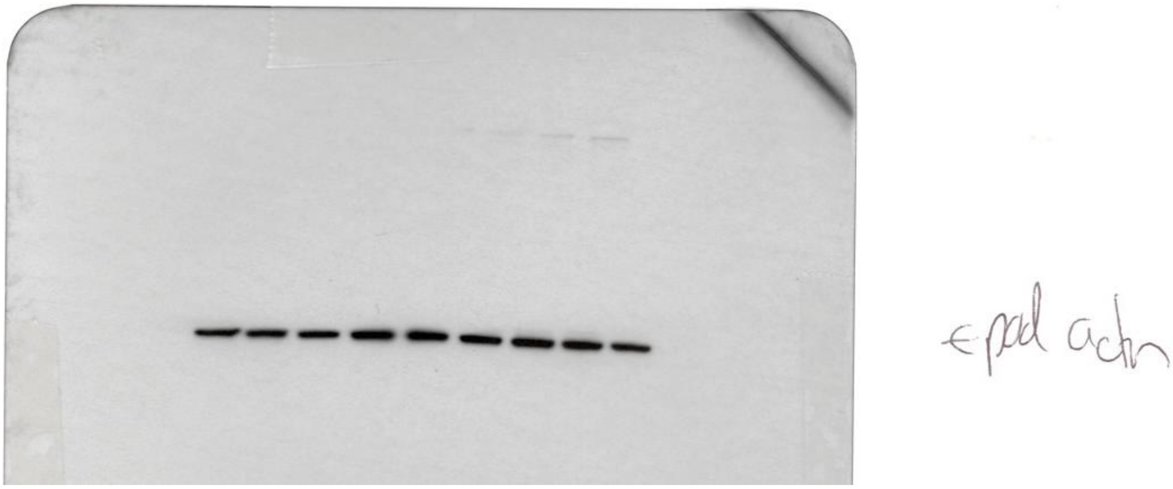

Original blot images for Caspase3 and beta actin on hPOD exposed to healthy or MN serum with or without C3aRA corresponding to blots in Figure 4. Synapotpodin was measured around 100 kDa; ROMO1 was measured around 10 kDa; Beta actin at 42kDa.

First 3 bands: Control (healthy serum); Central 3 bands: anti-PLA2R serum; right 3 bands: anti-PLA2R serum + C3aRA;

Synaptopodin

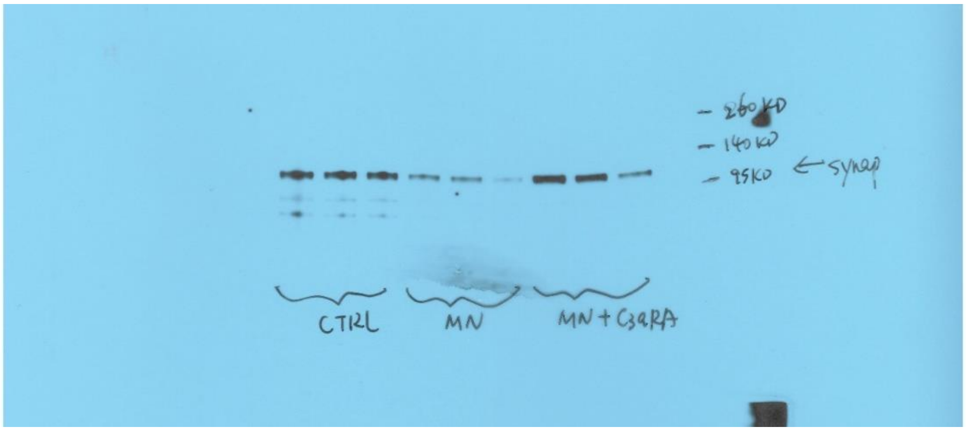

ROMO1

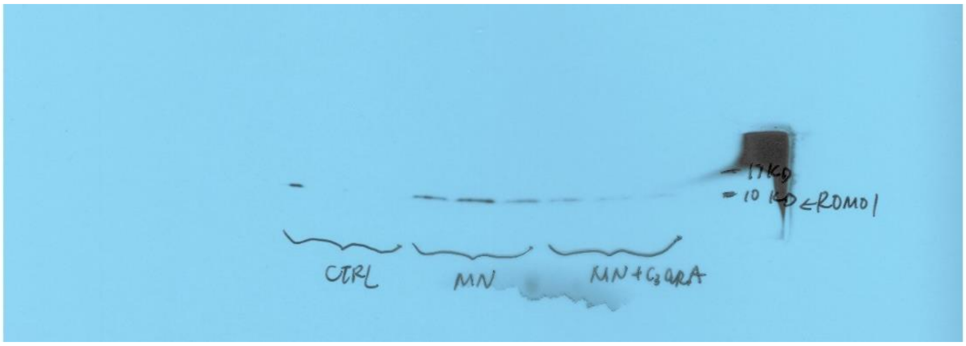

Beta actin for Synaptopodin and ROMO1

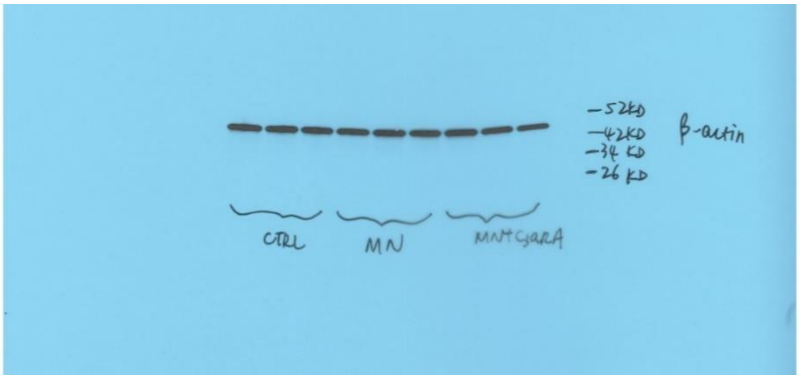

PLA2R, Caspase 3, C3aR1 and corresponding Beta actin

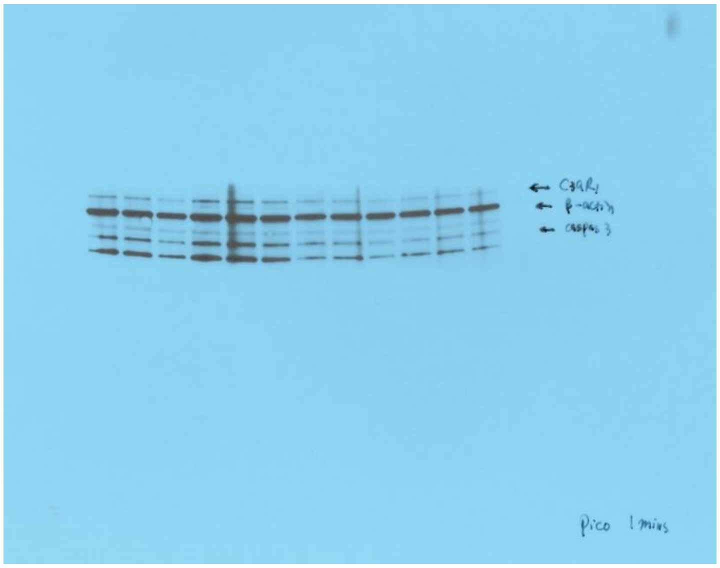

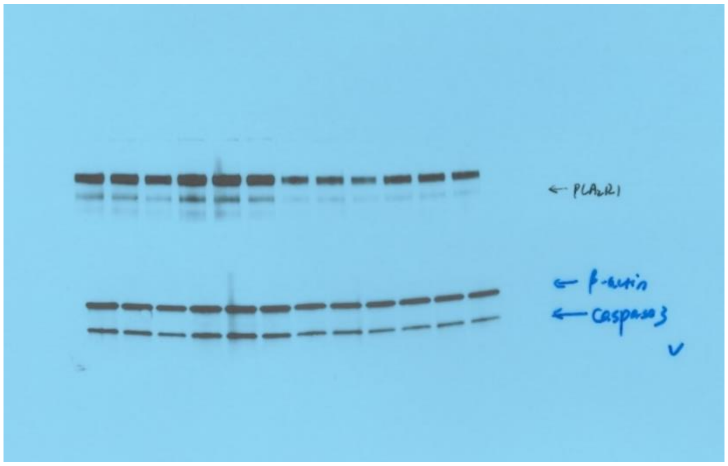

Full unedited Western Blotting gel image for figure S1G.
PLA2R1, human recombinant protein (positive control) and corresponding beta actin.

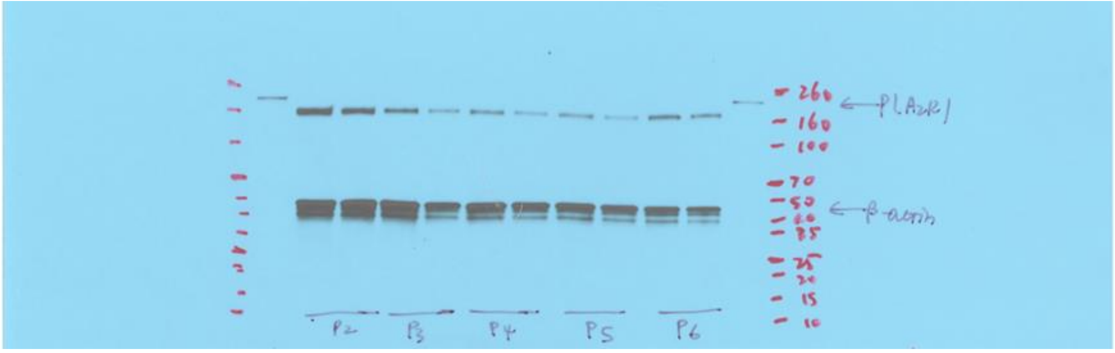

Full unedited Western Blotting gel image for figure S7A.
C5b9 and corresponding beta actin. First three lanes: Healthy serum; Central three lanes: MN serum; right three lanes: MN serum + protein S

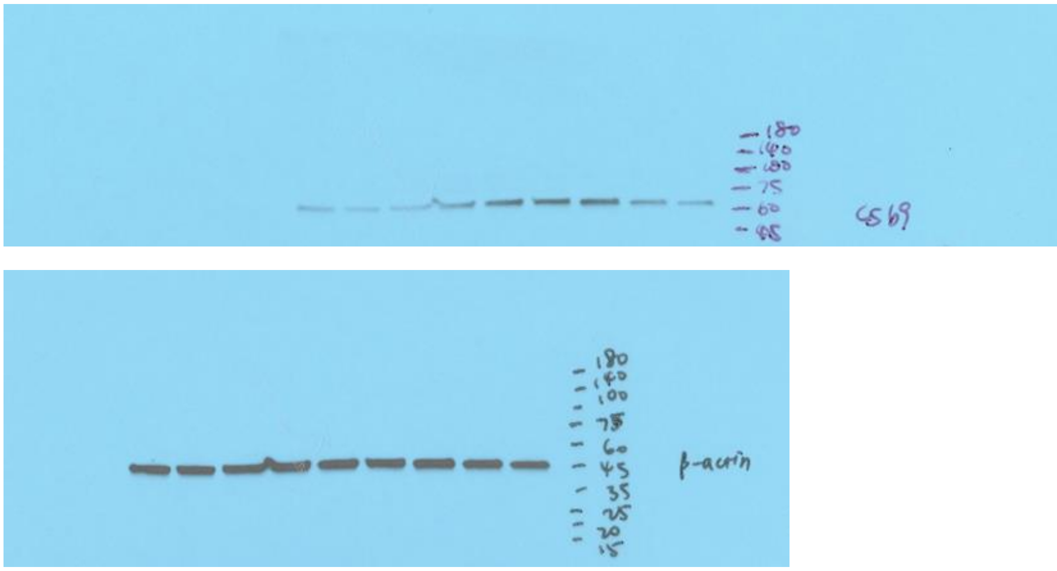

Full unedited Western Blotting gel image for figure S7D.
C3aR1, human recombinant protein (positive control) and corresponding beta actin.

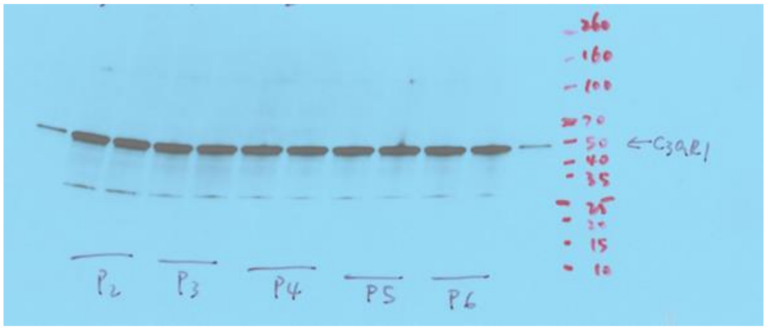

Full unedited Western Blotting gel image for figure S9.
C3aR1, Caspase-3 and corresponding beta actin.

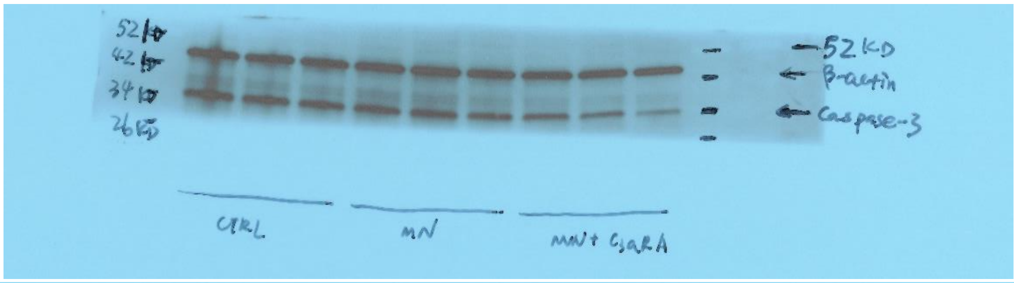

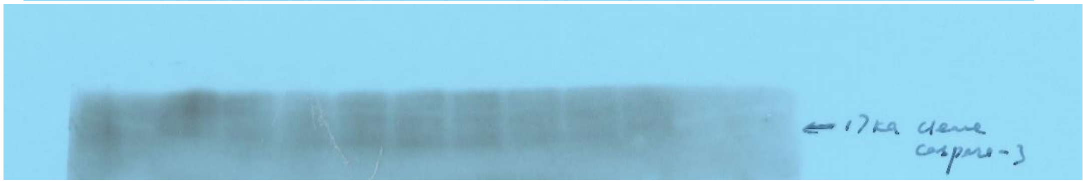

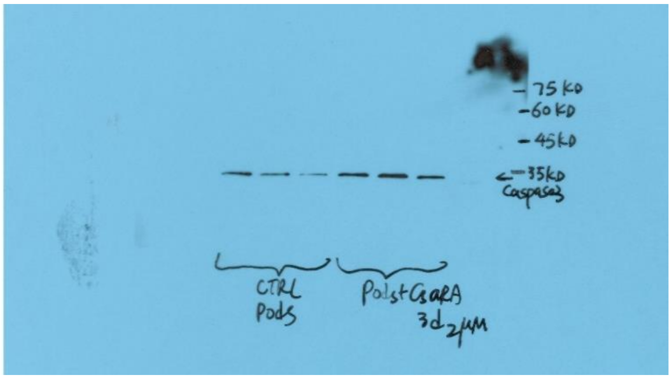

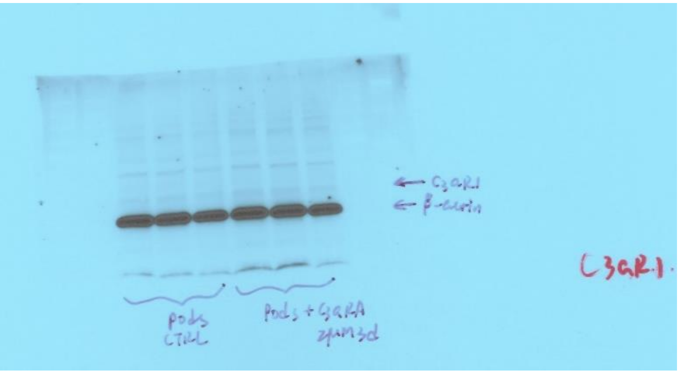

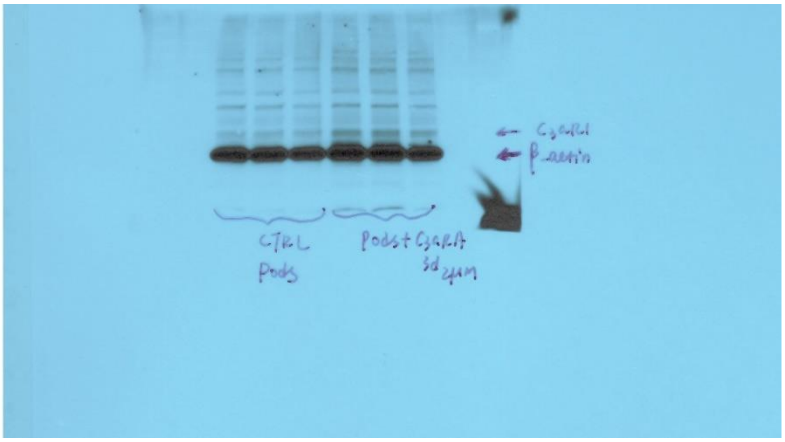

PLA2R and corresponding beta actin.

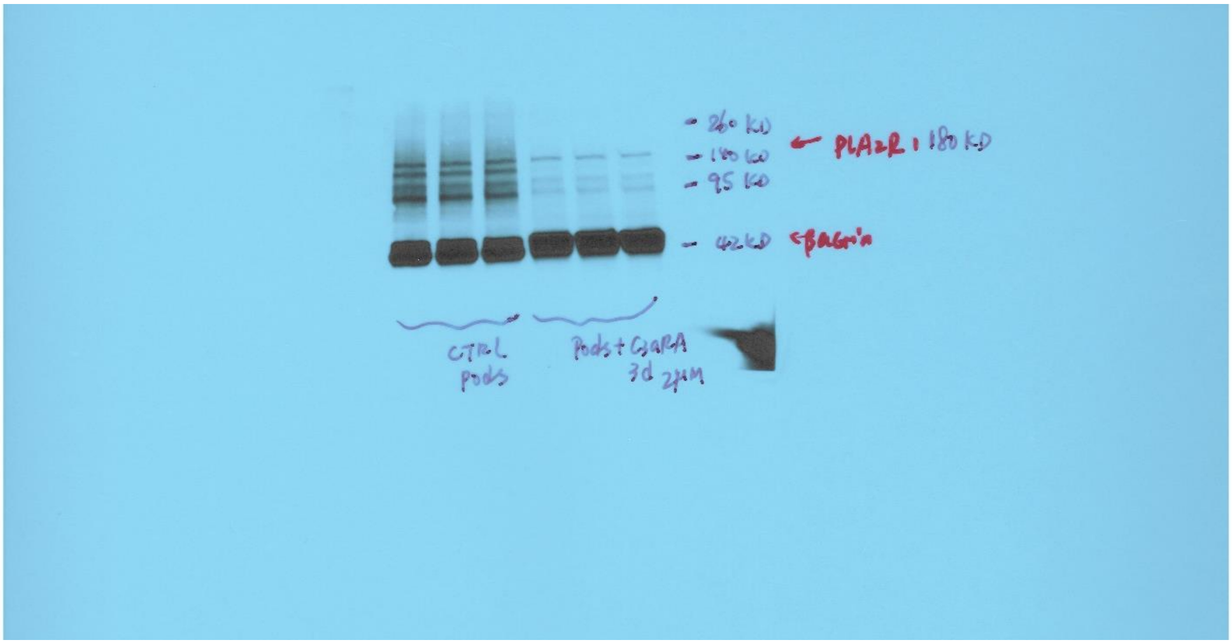

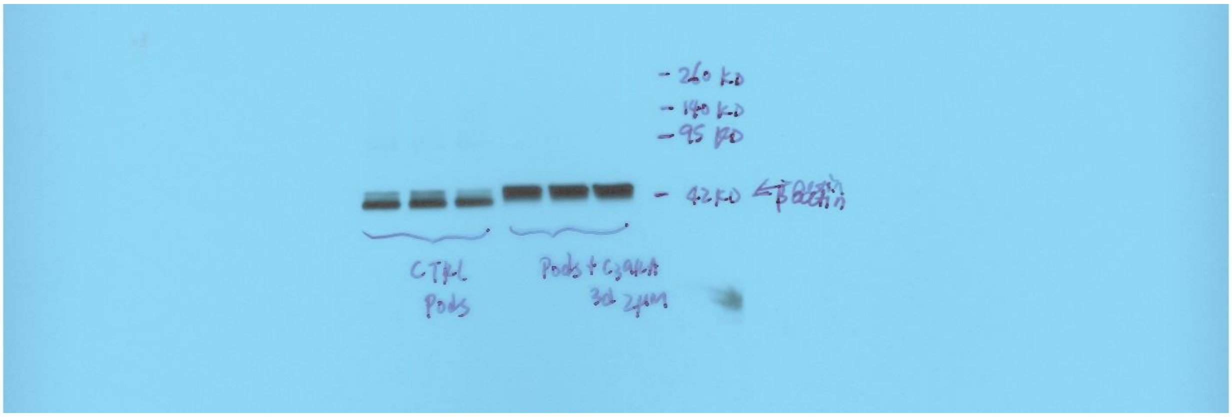

Full unedited Western Blotting gel image for figure S10.

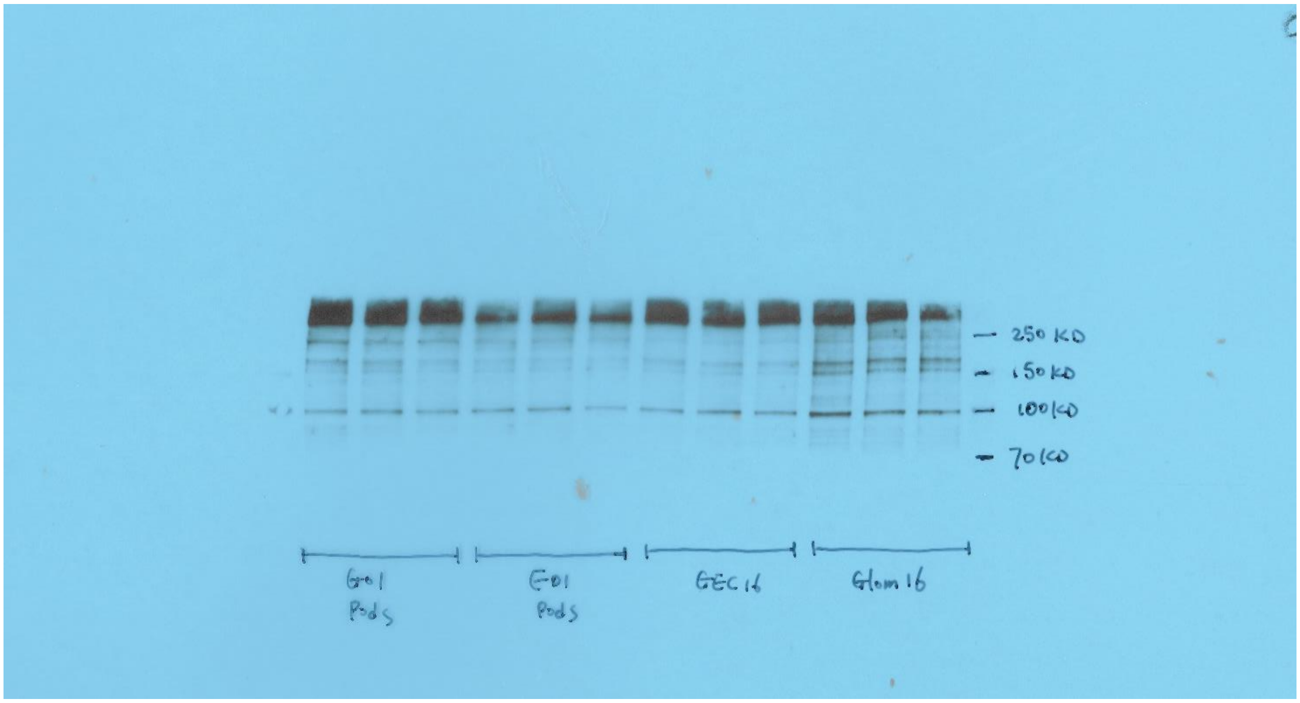

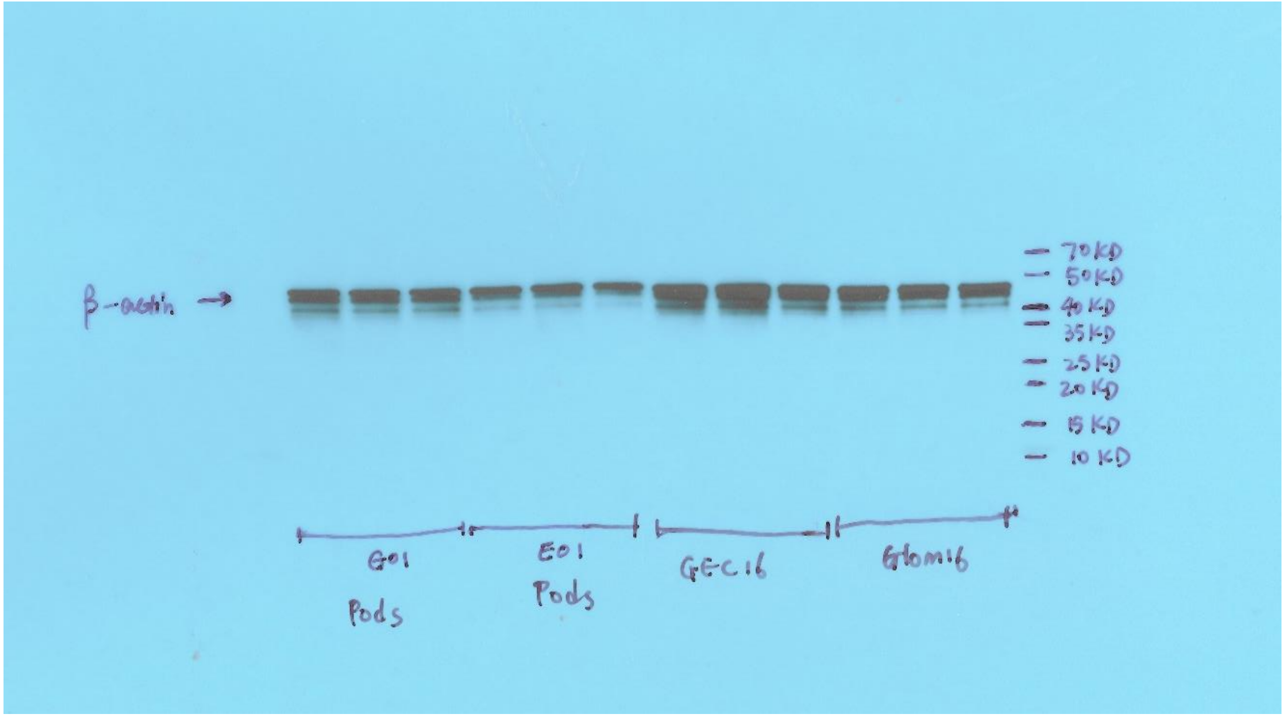

Supplement: Unedited blot and gel images [file jciinsight-9-172976-s071.pdf]
